# The Great Migration and African-American Genomic Diversity

**DOI:** 10.1371/journal.pgen.1006059

**Published:** 2016-05-27

**Authors:** Soheil Baharian, Maxime Barakatt, Christopher R. Gignoux, Suyash Shringarpure, Jacob Errington, William J. Blot, Carlos D. Bustamante, Eimear E. Kenny, Scott M. Williams, Melinda C. Aldrich, Simon Gravel

**Affiliations:** 1 Department of Human Genetics, McGill University, Montreal, Quebec, Canada; 2 McGill University and Genome Quebec Innovation Centre, Montreal, Quebec, Canada; 3 School of Computer Science, McGill University, Montreal, Quebec, Canada; 4 Department of Genetics, Stanford University School of Medicine, Stanford, California, United States of America; 5 Division of Epidemiology, Vanderbilt University School of Medicine, Nashville, Tennessee, United States of America; 6 International Epidemiology Institute, Rockville, Maryland, United States of America; 7 Department of Genetics and Genomic Sciences, The Icahn School of Medicine at Mount Sinai, New York, New York, United States of America; 8 The Charles Bronfman Institute for Personalized Medicine, The Icahn School of Medicine at Mount Sinai, New York, New York, United States of America; 9 The Icahn Institute for Genomics and Multiscale Biology, The Icahn School of Medicine at Mount Sinai, New York, New York, United States of America; 10 The Center for Statistical Genetics, The Icahn School of Medicine at Mount Sinai, New York, New York, United States of America; 11 Department of Genetics, Institute for Quantitative Biomedical Sciences, Dartmouth College, Hanover, New Hampshire, United States of America; 12 Department of Thoracic Surgery, Vanderbilt University School of Medicine, Nashville, Tennessee, United States of America; Georgia Institute of Technology, UNITED STATES

## Abstract

We present a comprehensive assessment of genomic diversity in the African-American population by studying three genotyped cohorts comprising 3,726 African-Americans from across the United States that provide a representative description of the population across all US states and socioeconomic status. An estimated 82.1% of ancestors to African-Americans lived in Africa prior to the advent of transatlantic travel, 16.7% in Europe, and 1.2% in the Americas, with increased African ancestry in the southern United States compared to the North and West. Combining demographic models of ancestry and those of relatedness suggests that admixture occurred predominantly in the South prior to the Civil War and that ancestry-biased migration is responsible for regional differences in ancestry. We find that recent migrations also caused a strong increase in genetic relatedness among geographically distant African-Americans. Long-range relatedness among African-Americans and between African-Americans and European-Americans thus track north- and west-bound migration routes followed during the Great Migration of the twentieth century. By contrast, short-range relatedness patterns suggest comparable mobility of ∼15–16*km* per generation for African-Americans and European-Americans, as estimated using a novel analytical model of isolation-by-distance.

## Author Summary

Genetic studies of African-Americans identify functional variants, elucidate historical and genealogical mysteries, and reveal basic biology. However, African-Americans have been under-represented in genetic studies, and relatively little is known about nation-wide patterns of genomic diversity in the population. Here, we study African-American genomic diversity using genotype data from nationally and regionally representative cohorts. Access to these unique cohorts allows us to clarify the role of population structure, admixture, and recent massive migrations in shaping African-American genomic diversity and sheds new light on the genetic history of this population.

## Introduction

The history of African-American populations is marked by dramatic migrations within Africa, through the transatlantic slave trade, and within the United States (US). By 1808, when the transatlantic slave trade was made illegal in the US, approximately 360,000 Africans had been brought forcibly into the US in documented voyages [1]. International and domestic slave trade continued to impose long-distance migration on enslaved African-Americans until the end of the Civil War, in 1865. By 1870, the US census reported 4.88 million “colored” individuals of which 90% lived in the South [2].

Despite the ban on slavery, economic and social perspectives for most African-Americans remained bleak. Better opportunities in the North (Northeast and Midwest) and West led millions of African-Americans to leave the South between 1910 and 1970 [3]. This demographic event known as the Great Migration profoundly reshaped African-American communities across the US [4]. Today, 45 million Americans identify as Black or African-American.

A history of slavery and of systemic discrimination led to increased social, economic, and health burdens in many African-American communities. Health disparities continue to be compounded by poverty, unequal access to care, and unequal representation in medical research. To reduce health disparity in research, many cohorts are currently being assembled to encompass more of the diversity within the US [5, 6]. These cohorts create opportunities in both medical and population genetics; they also require an understanding of genetic diversity within diverse cohorts. However, the large-scale migrations and incomplete genealogical records for African-Americans present a challenge for such an understanding. Previous studies have described the proportions of African, European, and Native American ancestries across individuals [7–13], the amount of diversity in sequence data [9, 14, 15] and inferred admixture models [12, 16, 17].

However, because previous cohorts were not representative of the general African-American populations, they provided limited information about population structure among African-Americans.

Here, we use cohorts including 3,726 African-Americans and a total of 13,199 individuals geographically distributed across the contiguous US to investigate nation-wide population structure among African-Americans. We first confirm and refine previous estimates of admixture proportions and timing in the population, and find significant differences in ancestry proportions between US regions. We then investigate relatedness among African-Americans and European-Americans through identity-by-descent analysis, and identify long- and short-range patterns of isolation-by-distance. We introduce quantitative models, incorporating both census data and fine-scale migration, to describe these isolation-by-distance patterns and infer migratory patterns in the population. Integrating quantitative models for admixture, relatedness information, and historical data, we identify ancestry-biased migrations during the Great Migration as a driving force for ancestry and relatedness variation among African-Americans. The analysis of geographically distributed cohorts through detailed mathematical modeling therefore helps us understand the distribution of genetic diversity in large cohorts and provides new insights into recent human demography.

## Results

### Cohorts

We analyzed data from three cohorts: (a) Health and Retirement Study [18] (HRS), with 1,501 African-Americans and 9,308 European-Americans sampled representatively across all US states, and including urban and rural regions; (b) Southern Community Cohort Study [19] (SCCS), including 2,128 African-Americans sampled within the southern US in rural locations; (c) 1000 Genomes Project cohort of 97 individuals of African ancestry from the southwest USA [20] (ASW). Genotypes were obtained on Illumina Human Omni 2.5M and Human 1M-Duo platforms, and joint analyses were performed on a common set of 553,795 high-quality SNPs (for detailed information, see Materials and Methods and S1 and S2 Tables).

### Admixture Patterns

**Fig 1:**
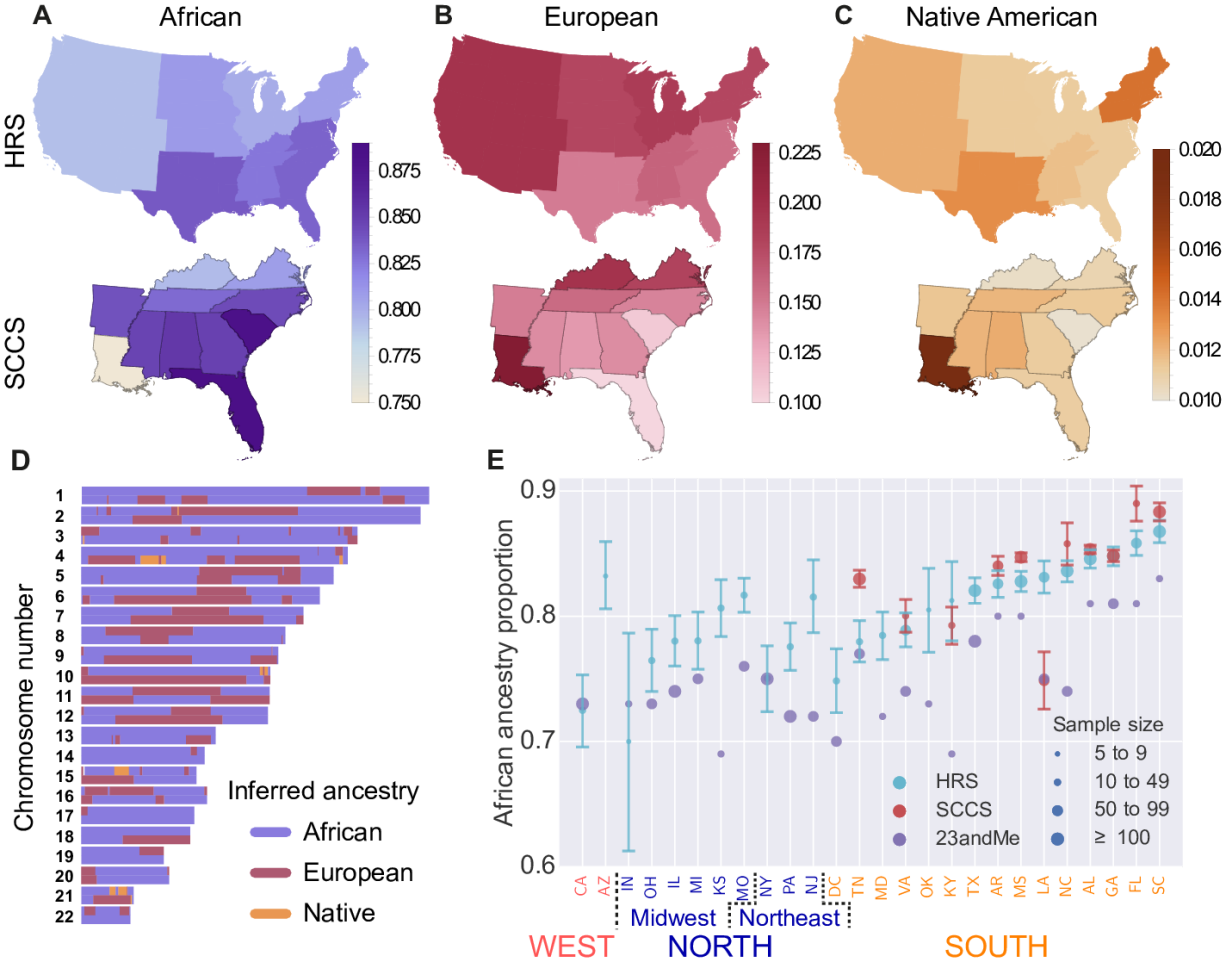
Inferred regional ancestry proportions for the HRS and SCCS cohorts: (A) African, (B) European, and (C) Native American ancestries. (D) Local ancestry assignment along the autosomes for an African-American individual from HRS. (E) Comparison of the African ancestry proportions in the HRS, SCCS, and 23andMe stratified by state. Error bars represent 68% confidence intervals derived using sample bootstrap and, thus, do not account for possible sampling biases. 23andMe proportions are from Ref. [12] and are reported for ease of comparison.

Individual genomes carry genetic material from multiple ancestral lineages, and each diploid locus derives ancestry from two distinct lineages. We used RFMix [11] together with 1000 Genomes Project panels from Africa, Europe, and Asia to identify the most likely continental ancestry at each locus for individuals in the cohorts (Fig 1D, S2 Fig and Materials and Methods). Here, continental ancestry is defined as the inferred location of the ancestral lineage prior to the advent of transatlantic travel. The overall proportion of African ancestry is substantially higher in the SCCS and HRS than in the ASW and the recently published 23andMe cohort [12] (Table 1).

**Table 1:** Inferred proportions of African, European, and Native American/Asian ancestry in three African-American cohorts with 95% confidence intervals based on sample bootstrap. These confidence intervals do not account for possible sampling biases.

Cohort	%African	% European	% Native American
SCCS	84.9, [84.47, 85.24]	14.0, [13.65, 14.43]	1.1, [1.07, 1.14]
HRS	82.1, [81.55, 82.66]	16.7, [16.16, 17.27]	1.2, [1.11, 1.27]
ASW	75.9, [72.96, 78.49]	21.3, [19.50, 23.20]	2.8, [1.53, 4.34]

The HRS cohort can be thought of as representative of the entire African-American population, while the SCCS focuses primarily on individuals attending community health centers in rural, underserved locations in the South. By contrast, the sampling for the ASW and 23andMe did not aim for specific representativeness, and the ascertainment in the 23andMe cohort might have enriched for individuals with elevated European ancestry (see Materials and Methods and discussion in [12]). In the HRS, average African ancestry proportion is 83% in the South and lower in the North (80%, bootstrap *p* = 6 × 10^−6^) and West (79%, *p* = 10^−4^) (Fig 1). Within the SCCS, African ancestry proportion is highest in Florida (89%) and South Carolina (88%) and lowest in Louisiana (75%) with all three significantly different from the mean (Florida *p* = 0.006, South Carolina *p* = 4 × 10^−4^, and Louisiana *p* < 10^−5^; bootstrap). The elevated African ancestry proportion in Florida and South Carolina is also observed in the HRS and in the 23andMe study [12], but Louisiana is more variable across cohorts (Fig 1E). As expected, European ancestry proportions largely complement those of African ancestry across the US.

**Fig 2:**
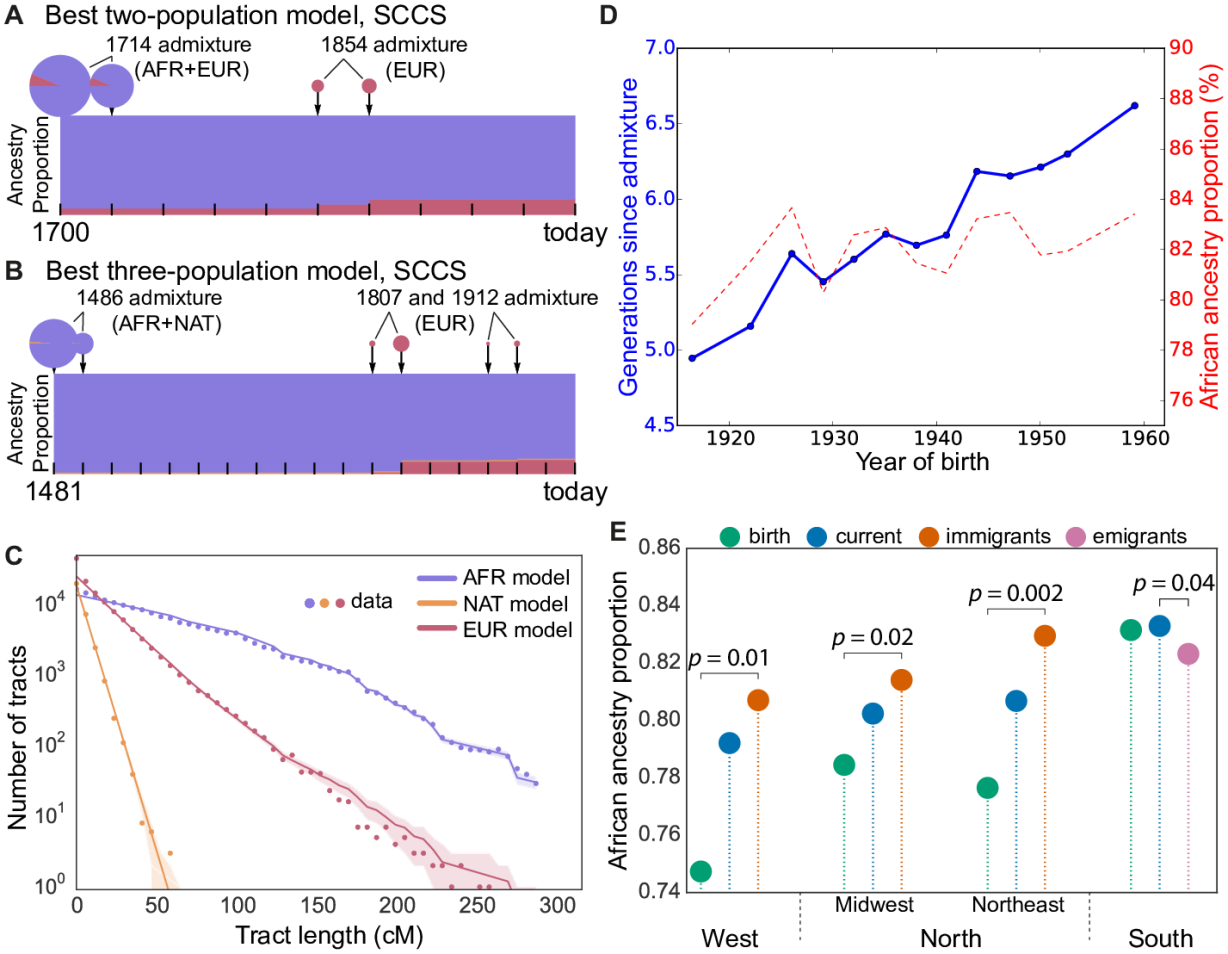
Admixture times and proportions of ancestral populations for SCCS in (A) the model with two pulses of admixture and (B) the model with three pulses of admixture. Because the model features a continuous time parameter but discrete generation times, a single pulse occurring at a fractional time contributes migrants to the two adjacent discrete generation times. African, European, and Native American ancestries are displayed respectively in blue, red, and yellow. Rectangles show the proportion of each ancestry at each generation. Pie charts represent migrations, with the size of the pie representing the amounts of migrants at a given generation and the sectors representing the proportion of migrants coming from each source population. (C) Distribution of continuous ancestry tract lengths (dots) compared with predictions from the best-fit model (lines) for SCCS. Points in the shaded area are within one standard deviation of the predicted result. Kinks in the distribution are due to the finite length of chromosomes [16]. (D) Inferred time to admixture and African ancestry proportions as functions of birth year in HRS African-Americans. (E) Proportions of African ancestry in African-Americans within the North, South, and West using region of birth, region of residence, and migration status; bootstrap *p*-values are calculated between disjoint sets of individuals.

Because recombination breaks down ancestral haplotypes over time (Fig 1D), the length of continuous ancestry tracts is informative of the time of admixture, with shorter tracts reflecting older admixture. We inferred the timing of admixture using Tracts [16], which fits a demographic history to the observed distribution of tract lengths (see Materials and Methods for details and S4 Table for confidence intervals). Because of the small number of Native American tracts, even a small amount of spurious Native American ancestry assignments can bias the inference. Thus, we first considered a model with two source populations: African and non-African. Assuming a single admixture event, we estimated the time of admixture onset *g*, where *g* = 1 means that the parents of the individual are the founders of the admixed population and that the current individual represents the first admixed generation. For HRS, we inferred a timing of *g* = 5.8 generations ago (S8 Fig). The estimated year of birth of the first admixed children is *T* = *T*_*s*_ − (*g* − 1)*τ*, where *T*_*s*_ = 1939.8 is the average year of birth of HRS individuals and *τ* is the generation time. Individuals born *τ* years earlier should be 1 generation closer to the onset of admixture. Correlating birth year and inferred admixture time within our cohort (Fig 2D), we inferred *τ* = 27.4 (*r*^2^ = 0.88, *p* = 10^−7^), which leads to an admixture year of 1808 (bootstrap 95% CI: [1805.5, 1810.4]). Note that 1808 represents the admixture time that best explains the data under the assumption of a single admixture event. The narrow confidence interval is, therefore, no guarantee that something exceptional occurred between 1805 and 1810. To investigate the role of modeling assumptions in admixture time estimate, we considered more general models.

A model allowing for two phases of European admixture outperforms the single-pulse model for HRS and SCCS (see Materials and Methods). In HRS, it suggests a first admixture event in 1740 (8.3 generations ago; bootstrap 95% CI: [1711.6, 1744.2]) and a second pulse, of approximately equal size, in 1863 (3.8 generations ago; bootstrap 95% CI: [1852.9, 1865.9]) (S8 Fig and Materials and Methods). Mean birth year in SCCS is *T*_*s*_ = 1946.9, supporting a single admixture event in 1802 (6.3 generations ago; bootstrap 95% CI: [1799.2, 1803.6]), or two events in 1714 and 1854 (9.5 and 4.4 generations ago; bootstrap 95% CIs: [1704.6, 1739.7] and [1849.8, 1868.7]) (Fig 2A, S8 Fig and S4 Table for confidence intervals). The two-pulse model remains a coarse simplification of the historical admixture process, but the data strongly supports ongoing admixture, predominantly before or around the end of the Civil War. This is consistent with historical accounts of “a marked decline in both interracial sexual coercion and interracial intimacy” [21] at the end of the Civil War (see also Ref. [22] and references therein).

The limited role of early 20th century admixture is further supported by the similarity in the inferred single-pulse time to admixture in all HRS census regions (between 5.4 and 6.2 generations ago, S11 Fig) and all cohorts, which is easily explained if most admixture occurred in the South prior to the Great Migration. The similar levels of African ancestry for all age groups within the HRS also support limited European admixture between 1930 and 1960 (Fig 2D). Importantly, more recent admixture is not represented in the SCCS and HRS cohorts; only two participants were born after 1970.

Time estimates point to admixture occurring when most ancestors to present-day African-Americans lived in the South. The regional differences in ancestry seen in Fig 1 are therefore unlikely to be caused by differences in recent admixture rates, and the large influx of migrants from the South would have strongly attenuated any earlier differences. An alternate explanation for regional differences in ancestry proportions is that individuals with higher European ancestry were more likely to migrate to the North and West during the Great Migration, a scenario we refer to as ancestry-biased migration.

To validate the ancestry-biased migration model, we compared ancestry proportions of HRS individuals according to their region of birth, residence, and migration status. European ancestry proportions in African-Americans who left the South (16.5%) is elevated compared to individuals who remained in the South (15.3%, bootstrap *p* = 0.04), confirming that ancestry-biased migrations continued at least to the mid-20th century. These migrants had substantially *less* European ancestry than African-Americans already established in the North (20.9%) and West (25.0%) (Fig 2E). Since the latter two groups received large contributions from the first wave of the Great Migration, this suggests that the proportion of European ancestry in first-wave migrants was higher than in the second wave—i.e., that there was stronger ancestry bias during the first wave of migration.

This change over time in ancestry-biased migration is consistent with historical accounts that southern African-American migrants to northern cities during the later stages of the Great Migration had darker complexion than North-born African-Americans (see [23], p. 179). The change could be explained by better social opportunities available to individuals with higher levels of European ancestry: Individuals with wealth and education were much more likely to migrate in the first wave of the migration (see [23], p. 167). Fig 2E shows that despite the ongoing ancestry bias, the migrations of HRS participants led to more uniform ancestry proportions across regions. Interestingly, the proportion of African ancestry among African-Americans *increased* in all four US regions between the time of birth and the time of survey of participants: The ancestry bias caused migrants to have levels of admixture between those of the South-born and North-born individuals. Their departures and arrivals both increased the regional African ancestry proportions.

Out of 1,491 non-Hispanic African-Americans in HRS, 11 individuals have more than 5% Native American ancestry. Within SCCS, this proportion is only 8 out of 2,128 individuals. The ASW cohort, with 8 out of 97 individuals above this threshold, is a clear outlier. The other 89 individuals, however, have similar amounts of Native American ancestry to the other studies. If we filter out individuals contributing more than 5% Native American ancestry from each cohort, the proportion of Native American ancestry in the remaining individuals is close to 1.1% in the SCCS, in all HRS census regions, and in the ASW. The filtered SCCS Louisianans have significantly more Native American ancestry (1.6%, bootstrap *p* = 2 × 10^−5^), and South Carolinians have less (0.09%, *p* = 2 × 10^−5^), than the mean Native American ancestry. We did not find a global correlation between European and Native American ancestry, except within Louisiana (S4 Fig).

A three-population admixture model accounting for Native American admixture confirmed the predominantly early, multiple-phase European admixture and suggested that Native American admixture occurred even earlier, consistent with previous findings [12]. Inferred dates of admixture onset are 1494 (bootstrap 95% CI: [1478.8,1516.0]) for the HRS (S9 and S10 Figs) and 1486 (bootstrap 95% CI: [1475.4, 1499.4]) for the SCCS (Fig 2B and 2C), as described in Materials and Methods. The presence of a small amount of spurious, short segments of inferred Native American ancestry could bias the inference toward these unrealistically early dates. The lack of longer Native American segments nevertheless suggests that most Native American ancestry in African-Americans results from contact in the early days of slavery (see, e.g., [24]). The three-population model suggests more recent European admixture dates than the two-population model, but with a higher proportion of migrants in the earlier migration. Finally, a three-population model with continuous European admixture provided qualitatively similar estimates to the two-pulse model, with an early onset of Native American admixture (1482) and European migration spanning the period between 1758 to 1887. Direct admixture between African-Americans and Native Americans is further supported by the observation that the proportion of Native American ancestry in HRS African-Americans (1.2%) is comparable to that in HRS European-Americans (1.5%). This proportion is therefore much higher than would be expected if the Native American contribution occurred through European admixture. Despite substantial disagreement as to the specific dates, all models agree on European admixture occurring predominantly prior to the Civil War.

Along the X chromosome in the HRS, we estimate 84.82% African ancestry, 12.89% European ancestry, and 2.29% Native American ancestry (bootstrap 95% CI [2.14%, 2.45%]). The higher proportion of African ancestry along the X compared to autosomes is consistent with previous studies [12, 17] and the historical record of early admixture occurring predominantly through coerced sexual interaction between European-American males and African-American females [21]. A model with a single pulse of admixture (as considered in [12]) applied to the present data suggests 28.6% Europeans among male contributors, but only 5.2% among female contributors. By contrast, it suggests almost no contribution from Native American males, and 3% from Native American females.

The US Census includes a separate category for Hispanic/non-Hispanic ethnicity. In HRS, 32 African-Americans have self-identified as Hispanics (of which only 10 are within the contiguous US). Hispanics often trace ancestry to regions colonized by Spain and Portugal, and where Native American populations contributed a higher proportion of the present-day gene pool compared than in the US. Genetic ancestry within this group is indeed distinct from the bulk of the non-Hispanic African-American population in at least two ways: elevated Native American ancestry and a higher genetic similarity to southern European populations (S5 and S6 Figs). The correlation between southern European and Native American ancestries also holds in individuals who do not self-identify as Hispanic, particularly in Louisiana (see Materials and Methods). Individuals with elevated Native American and southern European ancestry would not be identified by self-reported ethnicity or by genetic estimates of African/non-African ancestry, yet they may have distinct response patterns to medical tests [25, 26].

### Identity by Descent

The classical isolation-by-distance model predicts that genetic relatedness between individuals decreases as their geographic distance increases [27]. However, large-scale migrations can dramatically alter this picture [28]. To investigate the effect of recent migrations on patterns of genetic relatedness within African-Americans, we consider genetic segments that are identical-by-descent (IBD) between pairs of individuals. We focus on long IBD segments (*l* ≥ 18cM), which correspond to an expected common ancestor living within the last 8 generations (see Materials and Methods) and are therefore informative of recent demography.

**Fig 3:**
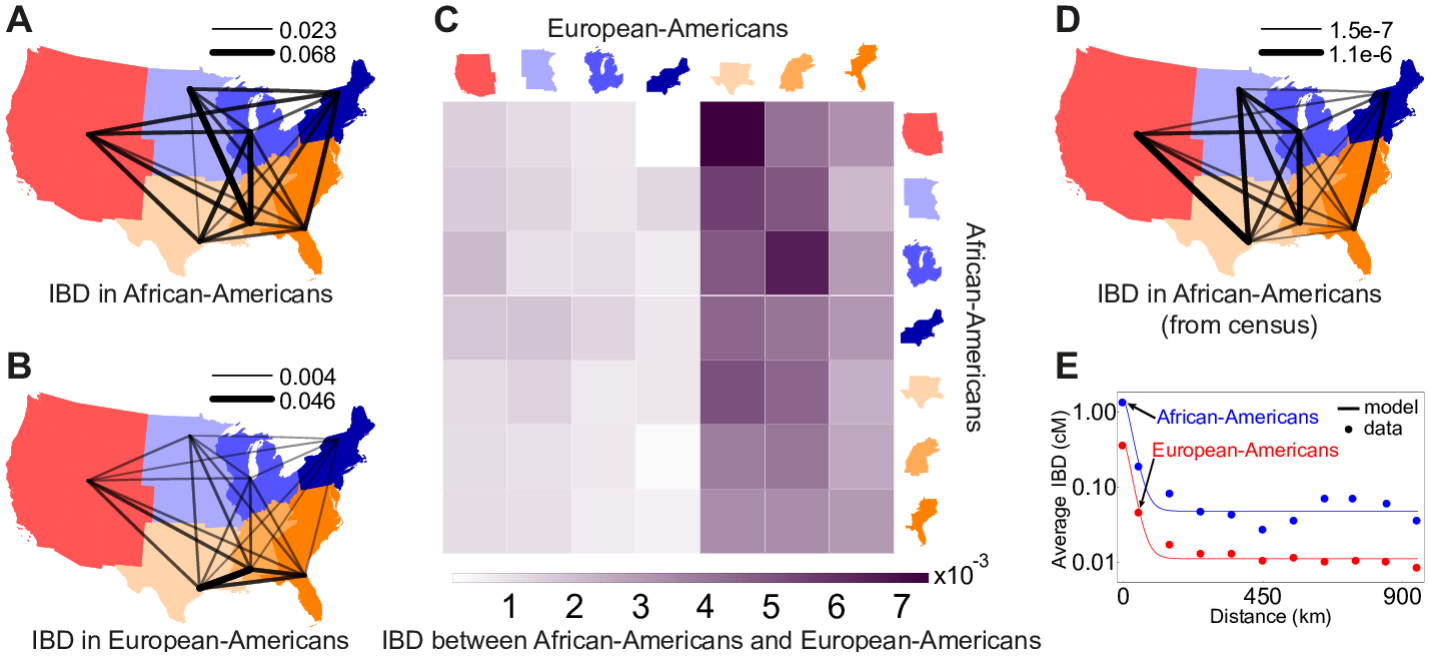
Pairwise genetic relatedness across US census regions among (A) African-Americans, (B) European-Americans, and (C) African-Americans and European-Americans. (D) Census-based prediction for African-Americans (see Materials and Methods). On each map, the line connecting two regions shows the average relatedness between individuals in those regions, and the thickness and opacity of the lines are on a linear scale between the minimum and maximum values shown above the map. Relatedness between regions with fewer than 10,000 possible pairs of individuals is not shown (see Materials and Methods for details). All numbers are in units of cM. (E) Decay of average IBD (shown in logarithmic scale) as a function of distance using IBD segments of length 18cM or longer from HRS (dots), compared to the analytical model (lines).

Fig 3A, 3B, S12 and S15 Figs show the mean pairwise relatedness among seven geographic regions in the US for African-Americans and European-Americans. Here, the relatedness of two individuals is defined as the total length of the genome shared through long IBD segments. These recent relatedness patterns differ markedly between African-Americans and European-Americans (compare Fig 3A and 3B): African-Americans exhibit a distinct enrichment in South-to-North relatedness along the main historical migration routes.

To compare these relatedness patterns with recent migration data, we used the 20th century US census data and a simple coalescent model to estimate the expected relatedness between geographic regions (see Materials and Methods). Census-based predictions (Fig 3D) are correlated with IBD-based observations (Fig 3A) if we consider non-identical pairs of regions (Mantel test *p* = 0.019). Limiting the comparison to the South-to-North and South-to-West relatedness, to capture migration routes specific to the Great Migration, yields *p* = 0.063 (using the 2010 region of residence) and *p* = 0.015 (using place of birth) (see Materials and Methods).

Fig 3C and S16 Fig show the relatedness between African-Americans and European-Americans. African-Americans across the US are more related to European-Americans from the South than to those from the North or West (bootstrap *p* < 0.0002). In addition, European-Americans from the South tend to be more related to African-Americans in the North than to those in the South (bootstrap *p* = 0.11). This increased relatedness with increased distance is unusual in population genetics, but is easily explained: The ancestry-biased migration is also a relatedness-biased migration. The reduced relatedness between northern European-Americans and African-Americans may also be reinforced by recent European migration, because the new migrants were more likely to settle in the North but were less likely to be related to African-Americans.

### Fine-Scale Isolation by Distance

Despite the unusual long-range relatedness patterns, identity-by-descent decays with distance within African-American communities in the South, reflecting isolation-by-distance (S19 Fig). To understand how migrations affect isolation-by-distance and identity-by-descent, we introduce a quantitative model taking into account a diploid population density *n* and spatial diffusion constant *D*. In short, the displacement between parental birthplace and offspring birthplace of individuals is modeled as an isotropic random walk; the distribution of the times *t* to the most recent common ancestor of two individuals separated by distance *R* is calculated under a coalescent model; and the amount of genetic material shared IBD given a common ancestor at time *t* is computed as in Ref. [29]. Under this model, we can calculate the expected fraction of genome shared IBD between two randomly chosen individuals separated by a distance *R*. If we consider only IBD segments of length in *ℓ* = [*l*_min_, *l*_max_] (in Morgans), we find

$${\text{E}}_{\ell }\left[f|R\right]=\frac{1}{16\pi nD}\left\{2 [K_{0}\left(\frac{R}{r_{\text{min}}}\right)-K_{0}\left(\frac{R}{r_{\text{max}}}\right)]+ [\frac{R}{r_{\text{min}}}\;K_{1}\left(\frac{R}{r_{\text{min}}}\right)-\frac{R}{r_{\text{max}}}\;K_{1}\left(\frac{R}{r_{\text{max}}}\right)]\right\}$$ (1)

where $r_{\text{min},\text{max}}=\sqrt{D/l_{\text{min},\text{max}}}$, and *K*_*α*_(*x*) is the modified Bessel function of the second kind [30] (see Materials and Methods).

Fig 3E shows the presence of a background level of IBD relatedness in both African-Americans and European-Americans even at long distances. This could be attributed to false positives in IBD calling, to relatedness originating prior to ancestral migrations from Europe and Africa into the Americas, or to a small amount of distance-independent migration. We account for these effects in our model by introducing an additional distance-independent (constant) term. Using IBD segments longer than 18cM, we estimate the background IBD for African-Americans and European-Americans in HRS to be *b*_AFR_ = 0.048cM and *b*_EUR_ = 0.011cM respectively (see Materials and Methods for details). We estimate population density *n*_AFR_ = 1.9km^−2^ and diffusion constant *D*_AFR_ = 63.5km^2^/generation for African-Americans across the US, and *n*_EUR_ = 7.6km^−2^ and *D*_EUR_ = 59.6km^2^/generation for European-Americans (Fig 3E). The ratio of European- to African-American inferred population density is therefore 3.9. According to the 2010 US Census, 13% of the total population have self-identified as “Black or African American alone” and 72% self-identified as “White alone”. The ratio of European- to African-American population size from the census is 5.5, in good agreement to our estimate above. Interestingly, the root mean squared displacement per generation, $2\;\sqrt{D\times 1\;\text{generation}}∼15-16\;\text{km}$, shows comparable local migration rates in European-Americans and African-Americans despite the different histories and population densities.

This root mean square (RMS) displacement is much less than the contemporary RMS parent-offspring dispersal in the US, estimated at 989km, but within the range of other modern human populations (2.6–300km) [31]. RMS displacement is heavily influenced by the largest displacement, and the latter study found approximately 27% of parent-offspring displacements in the US to be over the 1000km range. Such long-range migrations did not appear to leave a strong signature of isolation-by-distance in our IBD data and were captured by the uniform background term in our model. The RMS displacement in our model therefore does not account for such long-range migrations.

## Discussion

The history of African-American populations combines strong ties to place with large-scale migrations [4]. This comprehensive study shows the combined effects of fine-scale population structure, large-scale migrations, and admixture in shaping genetic diversity among African-Americans. Detailed models of genomic diversity recapitulate known historical events, such as the travel routes used during the Great Migration [4, 23] and the timing, amount, and geography of admixture between African, European, and Native ancestors [22, 24, 32–34]. They also quantify demographic effects that were less well characterized, such as ancestry-biased migration and the geographic patterns of relatedness among African-Americans. The observed ancestry-biased migrations of African-Americans suggest that the differences in social opportunity afforded to individuals with different levels of European ancestry at the time of the Great Migration [23] contributed to shaping the genetic population structure of contemporary African-Americans.

The observed patterns of relatedness have consequences for genetics research. Long IBD segments are often inherited from a recent common ancestor and are likely to carry shared but recent mutations. Such variants are more likely to be deleterious than older variants and are therefore prime targets for disease-mapping studies of rare traits [35]. Considering our analysis of long-range IBD sharing across the US, we expect rare monogenetic traits to be more often shared over long distances among African-Americans than among European-Americans, particularly along the routes of the Great Migration. Yet, their spatial distributions over short ranges should be as structured as in European-Americans.

Despite the overall correlation in regional admixture proportions among the SCCS, HRS, and 23andMe cohorts, significant differences remain in nation-wide and regional ancestry proportions. Such differences likely result from sampling biases that correlate with existing population structure through geography, urban/rural status, wealth, education level, and identity. Detailed sampling and sociodemographic modeling should therefore inform the design and analysis of large genetic cohorts that include African-Americans, as well as further efforts to understand the genetic makeup of African-American communities.

## Materials and Methods

### Ethics Statement

The use of these samples for the present study was approved by the IRB at McGill University and Stanford University, where the analyses were performed.

### Data

We used the genotype data of 12,454 individuals from the Health and Retirement Study [18] (HRS), genotyped on the Illumina Human Omni 2.5M platform, and of 2,169 African-American individuals from the Southern Community Cohort Study [19] (SCCS), genotyped on either Illumina Human Omni 2.5M or Human 1M-Duo platforms. The HRS cohort includes 1,649 individuals who self-identified as African-Americans (non-ambiguously in both HRS Tracker and dbGaP databases) and 10,432 individuals who self-identified as European-Americans. There are also 366 individuals labeled as “Others” whom we have not used in our main analyses (except in a PCA analysis, discussed below). The remaining 7 individuals have ambiguous, non-matching race identifiers in HRS Tracker and dbGaP, and we have, thus, excluded them from our analyses.

We performed comparisons with data from 23andMe [12] and from 97 individuals of African ancestry from the southwest USA (ASW) from the 1000 Genomes Project (at ftp://ftp.1000genomes.ebi.ac.uk/vol1/ftp/release/20130502/supporting/hd_genotype_chip/) [20]. The 23andMe cohort includes many African-American individuals and has been the subject of a detailed population genetic analysis [12], and the ASW cohort has been a reference African-American population in recent studies. However, these two cohorts were not meant to be representative of the US population. The 23andMe database has a complex ascertainment scheme, which may cause biases in ancestry and socioeconomic status. In particular, biases in regional representation and a small amount of survey response errors might lead to a lower European ancestry proportion. These possible biases are described in detail in [12]. Similarly, the ASW cohort was assembled from duos and trios with at least one Oklahoma resident, but with no attempt to reach geographic or demographic representativeness (Morris Foster, personal communication). For comparisons with the 23andMe study, we used the global ancestry proportions reported in [12], because the genotype data is not publicly available. The global ancestry proportions reported in the 23andMe study are calculated by first using their in-house local ancestry assignment pipeline and then aggregating the results across the genome, as described in detail in [12]; we employ a similar scheme, described below in detail.

### Data Merging and Quality Control

The HRS genotype data that we received had been already quality controlled, filtered, and phased. The SCCS cohort comprises data from 648 individuals in a breast cancer study (genotyped on Illumina Omni 2.5M platform) and 760 individuals in a prostate cancer study, 484 individuals in a lung cancer study, and 277 individuals in a colorectal cancer study (genotyped on Illumina Human 1M-Duo). All genotyped individuals were either cases or controls in their respective nested case-control studies. We converted the lung cancer dataset from human genome assembly hg18 to hg19 using the LiftOver utility from the UCSC Genome Bioinformatics Group and merged the four separate SCCS datasets into one using PLINK 1.9 [36]. During the merge process, we removed markers to which more than one name was assigned at the same position along a chromosome; removed markers with missing genotype calls; corrected unambiguous strand misassignments and removed ambiguous strand (mis)assignments; removed multi-allelic markers; and, finally, filtered the data for missing calls [37] first based on genotypes (PLINK argument --geno 0.0125) and then based on call rates per individual and minor allele frequency (PLINK arguments --mind 0.0125 --maf 0.01). The final SCCS dataset contains 2,128 individuals and 585,527 variants after these steps. We then used the same process to merge the HRS data with those of SCCS and ASW, resulting in a single dataset in PLINK format with 14,679 individuals and 553,795 variants. Performing a PCA on the data (pruning for LD leaves 77,902 markers), we found no batch effects (see S1 Fig). We then phased the merged data with SHAPEIT2 [38] (default arguments), and converted the output to PLINK format (while preserving the phasing information) using genetic map information from the 1000 Genomes Project data (at http://mathgen.stats.ox.ac.uk/impute/data_download_1000G_phase1_integrated_SHAPEIT2_9-12-13.html).

### Geographic Information

Geographic information in HRS is usually provided in the form of US census regions and divisions. We have used these locales in the ancestry analyses. ZIP code information for HRS study participants is available, but use of this data is restricted. We used zip code data only for the fine-scale spatial analysis of identity-by-descent relatedness. For SCCS, latitude and longitude coordinates of clinics were available. In the IBD analysis, we assigned the ASW individuals to the West South Central census division (see, e.g., https://catalog.coriell.org/1/NHGRI/Collections/1000-Genomes-Collections/African-Ancestry-in-SW-USA-ASW). In terms of geographic locations, we restrict our analyses to the census divisions in the contiguous United States (i.e., Pacific, Mountain, West North Central, East North Central, Middle Atlantic, New England, West South Central, East South Central, South Atlantic).

For the individuals in HRS, we only consider the ones born in the contiguous US who, at the time of sampling in 2010, also lived in the contiguous US; this reduces our sample size in HRS to 10,974 individuals of which 1,501 are self-identified African-Americans and 9,308 are self-identified European-Americans (with the remaining individuals being classified as “Others”). There are 4 additional individuals satisfying the geographic constraints above but who have discordant race identifiers in two different data files provided with the cohort data; these were removed from any downstream analysis. Among the unambiguous self-identified African-Americans and European-Americans mentioned above, there are respectively 10 and 427 individuals also self-identifying as Hispanics. The former 10 individuals are only included in our analysis of Hispanics status. In S1 Table, we summarize a few characteristics of the HRS African-American and SCCS cohorts, namely, the number of sampled individuals, the number of males and females, the number of Hispanics (if specified), and the locale.

African-American sample sizes in the New England and Mountain census divisions are small. We therefore merged the New England and Middle Atlantic divisions, and considered the Northeast census region as a whole. Similarly, we merged the Mountain and Pacific, and considered the West census division as a whole. The total number of geographic locales under consideration was therefore 7, namely, Northeast, Midwest consisting of 2 divisions, South consisting of 3 divisions, and West. We show in S2 Table the number of non-Hispanic individuals in our analyses separated by race and region of residence in 2010. The individuals are selected to have been born and to have lived within the contiguous United States at the time of sampling. These numbers are derived by combining the HRS, SCCS, and ASW cohorts, as described above.

### IBD Inference

We used GERMLINE [39] (arguments -err_hom 1 -haploid -bits 32 -w_extend) to infer IBD tracts of length 3cM or longer shared between individuals from the HRS, SCCS, and ASW cohorts. GERMLINE is prone to false positive IBD assignment, particularly at positions overlapping assembly gaps (see, e.g., [40]). It is therefore standard practice to filter out these regions [39, 40]. We developed a filtering strategy that improves on this practice by allowing the possibility of keeping long IBD tracts that span a troublesome regions, considering that GERMLINE is known to be more accurate for longer tracts [40].

We first count, for each genomic position, the number of overlapping IBD segments across all individuals. A chromosomal region is then marked as “forbidden” if the total number of IBD segments overlapping it is larger than a threshold, as follows. We determine a single background IBD count by comparing the total count for each position across the genome to the average count across the genome. We find that each genomic position is overlapped by approximately 15,000 IBD segments and, thus, take the threshold to be 25,000 to allow for some variation in the total number of IBD segments shared. Next, two forbidden regions will be merged as one if they are less than 0.1cM apart. IBD segments that overlap these forbidden regions are excluded from the downstream analysis unless they extend outside the forbidden regions by at least 3cM. In that case, we presume that there is sufficient evidence in the non-forbidden regions, and the segments are kept. After this filtering process, we are left with 8,664,251 IBD segments out of the total of 71,633,425, and a relatively uniform coverage of IBD across the genome.

### Regional Relatedness Using Genomic Data

Geographic information and inferred IBD segments were used to construct a relatedness metric between individuals and geographic regions within the cohorts. We first bin the IBD segments by length. The first bin contains segments of length between 3cM to 10cM, the second bin contains segments from 10cM to 18cM, and the last bin contains segments of length 18cM or longer. The latter bin corresponds to common ancestors living about 8 generations ago and is the focus of most of our discussion. Sorting the individuals by region and by African-American status within each region, we form two sparse relatedness matrices: L which contains the total IBD *length* shared between each pair of individuals, and N which contains the total *number* of shared IBD segments between each pair of individuals. The diagonal elements of L and N, which represent self-IBD, are set to zero by definition.

We next remove the contributions of closely related individuals from these matrices as follows. The HRS study has already identified 89 pairs of individuals having kinship coefficients greater than or equal to 0.1. To be consistent with the definition from HRS, we used PLINK to calculate kinship coefficients for SCCS and ASW individuals, labelling individuals with kinship coefficient of 0.1 or higher as related individuals. We find 22 related pairs among SCCS individuals, 62 related pairs among ASW individuals, and 1 related pairs between HRS and SCCS individuals (details below).

To see how geographic regions are associated based on the genetic relatedness of their inhabitants, we consider average pairwise IBD relatedness between regions [28]. The average pairwise relatedness *L* between two regions *R*_1_ and *R*_2_ is defined as the mean length of IBD segments shared between pairs of individuals, where one individual is from *R*_1_ and the other from *R*_2_. In addition, we consider the relationships between individuals of specific ancestry *S*_1_ and *S*_2_, each representing either African-American or European-American. Thus, the average total shared IBD length becomes

$$L_{\left(R_{1},S_{1}\right),\left(R_{2},S_{2}\right)}=\frac{\sum_{i,j}^{\prime }L_{ij}}{N_{\text{pairs}}}$$ (2)

where

- *i* and *j* are indices of two individuals who appear in L;
- the primed sum runs over relevant pairs (*i*, *j*) such that *i* < *j*, (*R*(*i*), *S*(*i*)) = (*R*_1_, *S*_1_) and (*R*(*j*), *S*(*j*)) = (*R*_2_, *S*_2_), where *R*(*i*) and *S*(*i*) denote the region and race status for individual *i*;
- *N*_pairs_ = *n*_1_ *n*_2_ if *R*_1_ ≠ *R*_2_ and *n*_1_(*n*_1_ − 1)/2 otherwise, with *n*_*i*_ being the number of individuals with attributes (*R*_*i*_, *S*_*i*_).

Using the metric defined above, we can calculate the pattern of relatedness between geographic locations among African-Americans, among European-Americans, and between African-Americans and European-Americans. The first two matrices are symmetric with respect to changes in the order of regions, whereas the last one is not.

### Visualization of Regional Relatedness

The following criteria were used for visualization of the IBD relatedness between regions. Due to the small number of sampled African-American individuals in the northern and western regions, the total number of IBD segments shared between these regions is small compared with that between other regions (see the bottom row in S14 Fig). Relatedness estimations are noisy for such pairs, and a scale that accommodates these noisy results would not allow for detailed comparison of less noisy results. Therefore, in Fig 3, S12 and S13 Figs, we did not draw the lines between any two *distinct* regions for which the total number of possible pairs of IBD individuals is less than 10,000 (e.g., notice the lack of connecting lines from West North Central to West). Since a significant number of the individuals in HRS are European-Americans, the number of IBD segments shared between European-Americans residing in any two regions is large enough to ensure the significance of the results, even when we restrict the analysis to the longest IBD segments (see the bottom row in S15 Fig).

### Regional Relatedness Using Census Data

We are interested in comparing the relatedness information derived from genomic data to those described in historical migration records, e.g., available from Integrated Public Use Microdata Series (IPUMS) [2]. Here, we describe a simple coalescent-based method to calculate a relatedness metric based on census data. Despite many simplifying assumptions, this metric is able to capture the dominant relatedness patterns originating from recent migration events and, therefore, provides a first-order model to understand relatedness patterns across the US.

We downloaded census data from 1900 to 1980 and extracted census year, census region, age, race, birth place, and weighted representation of each sample; the latter is the number of people in the population represented by the sampled individual. For any decade, we focus on the people in the age group of 20- to 30-year olds and consider the migrations of African-Americans and European-Americans separately. We assume a generation time of 30 years, thereby taking census years 1900, 1910, and 1920 as generation 3; 1930, 1940, and 1950 as generation 2; and 1960, 1970, and 1980 as generation 1. For each ancestry group, we construct a matrix whose elements $m_{ij}^{\left(g\right)}$ represent the number of migrations at generation *g* ∈ {1, 2, 3} from region *i* to region *j*; this matrix is highly asymmetric because of asymmetric nature of the migrations between geographical regions.

We now construct a heuristic census-based measure of relatedness between regions. Let us define $p_{i\to j}^{\left(g\right)}$ as the proportion of individuals in region *j* at generations *g* − 1 whose ancestors were in region *i* at generations *g*. In other words, the (*i*, *j*) element of the matrix *P*^(*g*)^ is

$$p_{i\to j}^{\left(g\right)}=\frac{m_{ij}^{\left(g\right)}}{\sum_{i^{\prime }}m_{i^{\prime }j}^{\left(g\right)}+m_{\text{out}\to j}^{\left(g\right)}}$$ (3)

where *g* ∈ {1, 2, 3} denotes the generation time of the ancestral population, $m_{ij}^{\left(g\right)}$ denotes the number of migrations from region *i* into census region *j* (as constructed above), and $m_{\text{out}\to j}^{\left(g\right)}$ is the number of migrants from outside of contiguous United States into the census region *j*. Had we not included migrations from outside the US into the mainland US, *P*^(*g*)^ would have been column-normalized (i.e., normalized with respect to the destination census regions).

A three-generation transition matrix can be constructed as

$$\bar{P}=P^{\left(3\right)}P^{\left(2\right)}P^{\left(1\right)}$$ (4)

where, by matrix multiplication of migration probabilities for all generations under consideration, ${\bar{P}}_{ij}$ takes into account all possible migration routes starting at region *i* and ending at region *j* that could have taken place in the span of these three generations.

To estimate genetic relatedness between different geographic regions, we further make the coarse assumption that population sizes were constant before 1910 and that populations were randomly mating. These parsimonious assumptions allow us to model the expected relatedness within regions using coalescent theory before the massive 20th century migrations. Neither assumption is expected to hold exactly, but the randomly mating, constant-population model is expected to capture the bulk of variation in the coalescence rate across regions.

Given ${\bar{P}}_{k,i}$ as the probability of a sampled individual from region *i* having an ancestor from region *k*, we define the census relatedness metric between regions *i* and *j* as

$$I_{ij}=\sum_{k}{\bar{P}}_{ki}{\bar{P}}_{kj}\frac{1}{N_{k}}$$ (5)

where *N*_*k*_ is the census population size of region *k*. Population size matters, because in larger populations, it is less likely that a given pair of individuals share a common ancestor. The number of common ancestors at each generation is approximately inversely proportional to *N*_*k*_, and therefore the expected recent shared ancestry is also approximately inversely proportional to *N*_*k*_. Thus, *I*_*ij*_ is proportional to the probability of two individuals from regions *i* and *j* having ancestors from (any) region *k* times the probability that these ancestors have a recent common ancestor within region *k*. Unlike $\bar{P}$ which is a directional metric, *I* is non-directional and symmetric and can be directly compared with the genetic relatedness matrix *L* in Eq (2), which was estimated using IBD data. The regional relatedness patterns derived using $\bar{P}$ and *I* are shown in S17 and S18 Figs.

### Significance Test for Genomic versus Census-Based Relatedness

To test the hypothesis regarding South-to-North migration corridors, we consider the matrix elements corresponding to relatedness between the three southern regions (South Atlantic, East South Central, West South Central) and the three northern ones (Northeast, East North Central, West North Central), forming a 3 × 3 matrix from the census data to be compared with the corresponding matrix from IBD data. To quantify the correlation between these two matrices, we use the Mantel test (which is a standard test of correlation between matrices) as follows. We perform 9! possible permutations on the elements of the matrix derived from the census data and calculate the Pearson correlation coefficient between the original IBD matrix and the permuted census matrix. We then accept or reject each permutation based on whether the calculated correlation coefficient is lower or higher than the correlation coefficient between the two original (non-permuted) matrices. The *p*-value is given by the ratio of the number of rejections to the total number of permutations (see main text for the numerical values). The *p*-value reported in the main text for the relatedness between South to North and West are derived by performing a random subset of 10^7^ permutations out of a total of 12! ones.

In addition to the tests above, we also perform a test using the region of birth of HRS individuals as their location, which roughly translates to the migrations during the first wave of the Great Migration. Given the average year of birth (1939.8) and the birth year distribution (S3 Table) in HRS, we only take, for consistency, generation 3 from the census data (see definition above) and write $\bar{P}=P^{\left(3\right)}$ as our overall directional relatedness matrix (compare with Eq (4) above). We then proceed as before to calculate the non-directional (symmetric) relatedness *I*. Given the new census-based prediction (using only *g* = 3 above) and the IBD relatedness pattern (using the region of birth), we perform a Mantel test, as described above, in order to find the correlation between the data and our prediction.

Even though Fig 3 only shows pairs of regions for which 10,000 possible pairs of individuals were available, the Mantel test procedure uses all pairs of regions regardless of the number of individuals they contain.

### Relatedness and Isolation-by-Distance

We wish to model the expected IBD relatedness between individuals in a spatially extended population. Our starting point is an idealized population living on a set of islands (or demes), with random mating within islands and migrations between the islands. We will consider a limiting example of a continuous population below.

We are interested in the probability that a genomic segment of given length, stretching across a specific locus, is shared identical-by-descent between two randomly selected individuals living on different islands. For identity-by-descent to occur, we need two events to happen: (a) lineages at that locus must have coexisted on one unknown island at some point in the past, and (b) these two geographically coexisting lineages must also have coalesced further in the past.

We measure time in generations and track lineages backwards in time. At each generation, we assume that the displacement between parental birthplace and offspring birthplace follows a random walk. Each lineage follows a random walk on the islands, with each step representing one generation back in time, connecting an individual to the ancestor from whom the locus is inherited. The lineages are then traced back until the time at which both ancestors coexist on the same island and coalesce in the most recent common ancestor in the next step back in time. We can, therefore, symbolically write the total probability of coalescence at a given generation as the probability of coexistence times the probability of coalescence, i.e.,

$$\text{Pr}\left(\text{coalescence}\right)=\sum_{\text{island}}\text{Pr}\left({\text{lineage}}_{1,2}\in \text{island}\right)\;\text{Pr}\left(\text{coalescence}|{\text{lineage}}_{1,2}\in \text{island}\right).$$ (6)

To derive the probability of coexistence, we first want to estimate the expected position of a lineage given its position in the past. Concretely, let **x**_0_ be the current location of an individual at *t* = 0. We would like to find Φ(**x**, *t*|**x**_0_), the probability that an individual’s lineage is on island **x** at *t* generations ago, given that it is currently on island **x**_0_.

By construction, the probability Φ(**x**, *t*|**x**_0_) takes into account contributions from *all* possible space-time paths that start at **x**_0_ and end at **x** at time *t*. For instance, a possible path is to arrive at **x** at *t*/2 and stay at that position until *t*, whereas another path is to arrive at **x** at *t*/3, leave **x** at the next step for a series of random walks to finally arrive at **x** again at *t*.

Consider a region of area Δ*A*_*i*_ that encompasses a deme with haploid population 2*n*(**x**_*i*_, *t*)Δ*A*_*i*_, where *n*(**x**_*i*_, *t*) is the effective diploid population density at position **x**_*i*_ and time *t* in the past. The probability that two lineages in Δ*A*_*i*_ coalesce in a given generation is

$$p_{\text{coal}}\left(x_{i},t\right)=\frac{1}{2n\left(x_{i},t\right)\Delta A_{i}}.$$ (7)

This expression does not consider the possibility of multiple coalescent events and is therefore appropriate only for a number of generations that is much less than the population size.

The discrete probability of two lineages having coexisted on the deme at **x**_*i*_ at time *t* in the past, given that they are a distance **R** apart (at **x**_0_ and **x**_0_+**R**) at present (at *t* = 0), is

$$p_{\text{coex}}\left(x_{i},t|R\right)=\Phi \left(x_{i},t|x_{0}\right)\Phi \left(x_{i},t|x_{0}+R\right).$$ (8)

Therefore, the total probability of having a common ancestor *t* generations ago in the discrete model is

$$p\left(t|R\right)=\sum_{i}\frac{\Phi \left(x_{i},t|x_{0}\right)\;\Phi \left(x_{i},t|x_{0}+R\right)}{2n\left(x_{i},t\right)\Delta A_{i}}.$$ (9)

To go from a discrete random walk to the continuous limit, we set Φ(**x**, *t*|**x**_0_)→*φ*(**x**, *t*|**x**_0_)Δ*A*, where *φ*(**x**, *t*) is now a continuous probability density. Thus, in this limit (with ∑_*i*_ Δ*A*_*i*_···→∫d^2^ **x**…), we get

$$p\left(t|R\right)=\int {\text{d}}^{2}x\;\frac{\varphi \left(x,t|x_{0}\right)\;\varphi \left(x,t|x_{0}+R\right)}{2n(x,t)}.$$ (10)

The continuous limit of a random walk process is the diffusion model. In this model, the probability density *φ*(**x**, *t*) of finding a lineage at an infinitesimal area d^2^ **x** centered around **x** at generation *t* in the past obeys the two-dimensional partial differential equation

$$\frac{\partial }{\partial t}\varphi \left(x,t\right)=\nabla_{x}\cdot [D\left(x\right)\nabla_{x}\varphi \left(x,t\right)]$$ (11)

where the diffusion coefficient *D*(**x**) encompasses the information related, in the discrete model, to probabilities of taking a step to an adjacent island or staying on the same island (for a discussion around the connection a random walk and a diffusion process, see http://ocw.mit.edu/courses/mathematics/18-366-random-walks-and-diffusion-fall-2006/). Solving for *φ*(**x**, *t*|**x**_0_) amounts to solving Eq (11) with initial condition *φ*(**x**, *t* = 0) = *δ*(**x** − **x**_0_) where *δ*(**x**) is the (two-dimensional) Dirac delta function.

For simplicity, we consider random walks with uniform probability of transitioning to any nearest-neighbor island, which translates to a constant (position-independent) *D* in the continuous model. We also assume that all islands have the same constant population size, leading to a population density which, on average, is constant in the continuous model.

Under these assumptions, we have

$$\varphi \left(x,t|x_{0}\right)=\frac{1}{4\pi Dt}\exp\left(-\frac{|x-x_{0}{|}^{2}}{4Dt}\right)$$ (12)

which, in turn, leads to

$$p\left(t|R\right)=\frac{1}{16\pi nDt}\exp\left(-\frac{R^{2}}{8Dt}\right)$$ (13)

with *R* = |**R**|.

Following Palamara *et al.* [29], we approximate the expected fraction of the genome shared through segments in the length range *ℓ* = [*l*_min_, *l*_max_] (in units of Morgans) as

$${\text{E}}_{\ell }\left[f|R\right]=\int_{l_{\text{min}}}^{l_{\text{max}}}\text{d}l\int_{0}^{\infty }\text{d}t\;p\left(l|t\right)p\left(t|R\right)$$ (14)

with *p*(*l*|*t*) = (2*t*)^2^*l* exp(−2*tl*) the probability density of an IBD segment of length *l* (in units of Morgans) spanning the locus shared by the two randomly chosen individuals whose lineages coalesce *t* generations ago. Performing the integrals above leads to the following closed form solution for the expected fraction of the genome shared as a function of spatial separation

$${\text{E}}_{\ell }\left[f|R\right]=\frac{1}{16\pi nD}\left\{2 [K_{0}\left(\frac{R}{r_{\text{min}}}\right)-K_{0}\left(\frac{R}{r_{\text{max}}}\right)]+ [\frac{R}{r_{\text{min}}}\;K_{1}\left(\frac{R}{r_{\text{min}}}\right)-\frac{R}{r_{\text{max}}}\;K_{1}\left(\frac{R}{r_{\text{max}}}\right)]\right\}$$ (15)

where *K*_*α*_(*x*) is the modified Bessel function of the second kind [30], and $r_{i}=\sqrt{D/l_{i}}$ with *i* ∈ {min, max}. Expanding for small *R*, we find ${\text{E}}_{\ell }[f|R]≃\frac{1}{16\pi nD}[\text{ln}(l_{\text{max}}/l_{\text{min}})-(l_{\text{max}}-l_{\text{min}})R^{2}/4D+O(R^{4})]$. We can use Eq (15) to approximate the amount of IBD in a finite chromosome of length *L*_*c*_ by setting *l*_max_ = *L*_*c*_ in Eq (15). This yields *E* [*f*_*c*_|**R**]≡*E*_[*l*_min_, *L*_*c*_]_ [*f*|**R**]. We come back to this approximation at the end of this section.

The total length of shared IBD tracts across all chromosomes, *L*, between a random pair of individuals, therefore, becomes

$$\text{E}\left[L|R\right]=\sum_{c=1}^{22}L_{c}\;\text{E}\left[f_{c}|R\right].$$ (16)

This quantity can be directly compared with that calculated from the IBD data to estimate the parameters of the model. Technically, this model makes two important relatively coarse approximations. First, in Eq (14), we have integrated from *t* = 0, even though coalescence from time *t* = 0 to *t* = 1 is not allowed. Second, when considering finite chromosome, Access to the exact location of clinics at which the SCCS cohort was sampled allows us to investigate the relation between IBD relatedness and spatial distance. Having inferred possible IBD segments using GERMLINE, we calculate, for each pair of individuals from SCCS, the total length of shared IBD and the distance between the clinics in which they were sampled. We make the underlying assumption that each individual lives close to the clinic at which he or she was sampled. Each pair is then placed, based on the distance between the two individuals, into one of the length bins in {[0, 1), [1, 101), [101, 201), [201, 301), …} (all numbers in kilometers). The first length bin, [0, 1), contains individuals sampled at the same clinic. For each bin, we calculate the average pairwise IBD length (the sum of the IBD lengths of all pairs divided by the total number of points in the bin) and assign it to a distance equal to the midpoint of the bin (e.g., for the length bin [1, 101), the assigned distance is 51km). The result is shown in S19 Fig.

Apart from the expected decay of relatedness with distance, we also notice the presence of a constant background IBD. This background IBD is larger for shorter IBD segments. As mentioned in the main text, this could be attributed to two possible factors: (a) GERMLINE has a higher false positive detection rate for shorter IBD segments [40] which is independent of the distance between individuals, or (b) shorter IBD segments, being much older on average, reflect history prior to migrations from Europe and Africa into the Americas. Since this relatedness patterns extends over long distances with little evidence for decay, we suppose that it is either due to false positives, or that there was enough mixing in the travels into the Americas that present-day proximity is a relatively poor proxy for the proximity of ancestors prior to transatlantic travels. In either case, the background IBD can be modeled by adding a constant term to our model in Eq (16), representing the expected fraction of the genome shared IBD by individuals over long distances.

The parameters to be inferred in this model are the haploid population density *n*, the diffusion coefficient *D*, and background IBD *b*. By fitting the SCCS African-American IBD data for the 18cM case (corresponding to the most recent sharing events), we find the estimated values *b*_18_ = 0.0389cM, *n*_18_ = 2.8km^−2^, and *D*_18_ = 88.6km^2^/generation. The root mean squared displacement for African-Americans in the South is thus estimated, using the IBD data from SCCS, to be 18.8km. We can use the population density and diffusion coefficient derived above to predict IBD decay for IBD segments of different lengths and estimate the background IBD for the other two cases (bins with segments of length 10cM or longer and with segments of length 3cM or longer), finding *b*_10|18_ = 0.120cM and *b*_3|18_ = 0.546cM. The resulting fits show good agreement with the data, as shown in S20 Fig.

The current model of isolation-by-distance makes two approximations in addition to the assumptions of a uniform, random-mating population. First, following Ref. [29], we approximated a discrete-generation model with a continuous-time model, as shown by the time integral in Eq (14). The integral’s lower bound at *t* = 0 suggests that close relatives are included in the model. Second, we assumed an infinite-genome approximation for *p*(*l*|*t*), as derived in Ref. [29], and accounted for finite-genome effects by setting *l*_max_ = *L*_*c*_, noting that a shared IBD tract on chromosome *c* can be at most of length *L*_*c*_. However, to properly account for finite-genome effects, it would be preferable to consider the IBD segments in the infinite-genome scenario and derive their appropriate distribution using a sliding ‘window’ to represent a chromosome of finite length [16]. To verify that our results are robust to these approximations, we computed $\tilde{p}(l|L_{c},t)$ using the finite-genome approach of [16] and performed the proper integrals, from *t* = 1 onward, numerically. This was considerably more computationally intensive, but we found that the these corrections lead to results that are qualitatively very similar to what we have derived using the simpler approach described in Eq (16), with the effective population density *n*_18_ ≃ 2.4km^−2^ and the diffusion coefficient *D*_18_ ≃ 88.4km^2^/generation for the SCCS cohort.

### Effect of Phasing Errors and Ascertainment Bias

Our IBD-based results could be sensitive to computational phasing errors which break up IBD tracts into shorter ones. To assess the overall effect of these errors, we used RFMix to perform phase correction on a subset of the data, used this output for IBD calling with GERMLINE, and recalculated regional relatedness patterns. We then compared these new patterns with those obtained from the same subset of data without a phase correction step. We did not observe any significant difference in relatedness values between geographical regions across the US. For the isolation-by-distance scenario, we expect that the breaking of IBD tracts would lower the overall relatedness uniformly, thereby we expect to have underestimated the densities and, similarly, overestimated the displacements by a small margin.

We expect the ascertainment bias to have negligible effect on our analyses, given that our results are based on information obtained from long haplotypes as opposed to that obtained from summary statistics based on single SNPs (e.g., allele frequency) which are more likely to be sensitive to SNP ascertainment scheme [41].

### Expected *T*_MRCA_ Given Length of IBD Segments

For reference, we derive the expected generation time to the most recent common ancestor (MRCA), given an IBD tract of certain length. The probability density of having an IBD segment of length *l* (in units of Morgans) spanning a chosen marker (denoted by *ζ*) inherited from a MRCA living *g* generations ago (assumed continuous for simplicity) is [29]

$$p\left(l|g\right)={\left(\frac{2g}{1\text{M}}\right)}^{2}l\exp\left(-\frac{2g}{1\text{M}}\;l\right).$$ (17)

In the continuous limit to the Wright-Fisher model, given the shared locus *ζ*, the probability of having a MCRA *g* generation ago is

$$p\left(g\right)=\frac{1}{N}\;e^{-g/N}$$ (18)

where *N* is the (constant) effective haploid population size. Therefore, given the length *l* of an IBD tract (in units of Morgans), we use Eqs (17) and (18) to find the expected value for the generation time of the MRCA

$$\text{E}\left[g|l\right]=\int_{0}^{\infty }g\;p\left(g|l\right)\;\text{d}g=\int_{0}^{\infty }g\;\frac{p(l|g)p(g)}{p(l)}\;\text{d}g=\frac{\int_{0}^{\infty }g\;p\left(l|g\right)p\left(g\right)\;\text{d}g}{\int_{0}^{\infty }p\left(l|g\right)p\left(g\right)\;\text{d}g}≃\frac{3}{2(l/1\text{M})}$$ (19)

where we have assumed that the haploid population size *N* ≫ 1 in the last step.

### Local and Global Ancestry Analysis

After the phasing process (discussed previously), we used RFMix [11] with arguments PopPhased --skip-check-input-format for local ancestry inference along the genome. We used available parents among the trios in the Southern Han Chinese (CHS), Yoruba in Ibadan, Nigeria (YRI), and Utah Residents (CEPH) with Northern and Western European Ancestry (CEU) populations from the 1000 Genomes Project (at ftp://ftp.1000genomes.ebi.ac.uk/vol1/ftp/phase1/analysis_results/supporting/omni_haplotypes/) as a reference panel, comprising 50 CHS, 97 YRI, and 91 CEU individuals. We extracted the intersecting set of SNPs between our merged dataset and the three reference populations mentioned above, which we used as the input to RFMix. RFMix assigned continental ancestry of each marker in each sample to either CHS, YRI, and CEU, which we interpret as Native American/Asian, African, and European respectively. The local ancestry calls from RFMix for the SCCS are available from the Southern Community Cohort Study cohort through the Online Request System (ORS).

We used the local ancestry estimates obtained from RFMix to calculate global ancestry proportions for the HRS, SCCS, and ASW cohorts by dividing the total length of all tracts assigned to an ancestry (African, European, and Native American/Asian) to the total length of all assigned tracts (see S2 Fig).

For the X chromosome, a supervised run of ADMIXTURE with *K* = 3 reference populations (YRI representing African ancestry, CEU representing European ancestry, and CHS representing Native American/Asian ancestry) provided the ancestry breakdown shown in S3 Fig.

### African-Americans of Hispanic Background

We performed a supervised *K* = 4 run of ADMIXTURE [42] on African-Americans from HRS, SCCS, and ASW, with the YRI, CHS, GBR, IBS cohorts from the 1000 Genomes Project used as the reference populations representing African, Native American/Asian, northern European, and southern European ancestral populations. Pruning for LD was performed based on the recommendations of the authors of ADMIXTURE (PLINK arguments --indep-pairwise 50 10 0.1). The mean ancestry proportions for African-Americans in HRS, as estimated by ADMIXTURE, are 81.583% for African, 17.333% for European (southern and northern combined), and 1.083% for Native American, in very good agreement with those derived using local ancestry estimates of RFMix (see main text). In comparison, the ancestry proportions for the ASW cohort are 75.726% for African, 21.881% for European (southern and northern combined), and 2.394% for Native American.

S5 Fig depicts the ancestry estimates for African-Americans in the ASW, HRS, and SCCS cohorts respectively, sorted by Native American proportions (shown in yellow). The top panel shows that ASW individuals with higher proportion of southern European ancestry (shown in green) tend to also have a higher proportion of Native American ancestry, and this pattern is repeated in the other two cohorts. This is especially true for HRS African-Americans who have self-identified as Hispanics (marked by the small black arrows in the middle plot). This correlation is also apparent in S6 Fig, which shows the proportion of southern European ancestry within the total European ancestry versus the Native American ancestry for HRS African-Americans. The correlation is particularly clear for self-identified Hispanic individuals. Note the presence of individuals who have *not* self-identified as Hispanics but have high proportions of both southern European and Native American ancestries. Moreover, SCCS African-Americans from Louisiana exhibit a similar pattern, as depicted by the black dots in S6 Fig.

### Quality Control of Local Ancestry Inference

To ensure that the inferred Native American ancestry reflects the true Native American ancestry, and not mis-assignment of European or African ancestry segments, we performed simulations based on a two- and a three-population admixture model. In both cases, we generated ancestry tracts for 50 admixed diploid genomes in a forward Wright-Fisher model with a single pulse of admixture 8 generations ago.

For the two-population admixture model, the ancestry proportions in the simulated individuals were 74.96% African and 25.04% European. We copied genotypes from one YRI sample into African ancestry segments and one TSI sample into the European segments (both samples from the 1000 Genomes Project) to generate 100 haploid chromosome 1’s. Each chromosome 1 was generated using a distinct source chromosome in the YRI and TSI population. We then inferred the ancestries of the individual *i* (corresponding to haplotypes 2*i*−1 and 2*i*) with panels composed of samples chosen from 91 CEU, 50 CHS, and 96 YRI, ensuring that the individual from whom the genotypes were copied was not used in the reference panel. We inferred 74.96% African, 24.95% European, and 0.09% Native American ancestry.

For the three-population admixture model, we simulated a sample of 100 haploid chromosomes with 80.9% YRI, 18.2% TSI, and 0.91% JPT ancestry, using the same method described above. In this case, the inferred proportions were 80.9% African, 18.2% European, and 0.94% Native American. These results are consistent with previous estimates of false assignment using a similar pipeline [11].

We also considered whether the amount of Native American ancestry in real samples correlated with the amount of European ancestry. If European segments are more likely to be misinterpreted as Native American, we would expect a positive correlation between inferred Native American and European proportions. Conversely, if the increased diversity in African segments led to higher rates of misidentification as Native American ancestry, we’d expect the correlation to be negative. The relation between Native American ancestry and European ancestry within SCCS is shown in S4 Fig. Within the southern states, only Louisiana shows a significant correlation. The lack of global correlation between European and Native American ancestry helps support the correctness of the inference.

Finally, we also compared global ancestry proportions inferred by RFMix and by ADMIXTURE (in supervised mode) and found an extremely high correlation between the estimates from the two methods, as shown in S7 Fig.

### TRACTS and Timing Estimates

To infer time of admixture between ancestral populations and to identify migration models that give rise to the observed genome-wide patterns of ancestry, we use Tracts [16]. We excluded for this analysis HRS African-Americans from non-mainland US (96 individuals), African-Americans with self-reported Hispanic ethnicity (32 additional individuals), and one additional African-American who was listed as “White, non-Hispanic” in HRS Tracker but as “African-American” in dbGaP. All individuals were kept in the other cohorts. Optimization was performed for 6 models in each cohort: 2 two-population models, and 4 three-population models. Confidence intervals were then calculated.

#### Two-population admixture models

The first two-population model, pp, consists of a single pulse of discrete admixture between an African (AFR) and a non-African (NONAFR) population. The second model, pp_xp, considers a pulse of admixture from each population, followed by a second pulse of admixture from the NONAFR one. In the model nomenclature, migration events are described by strings separated by underscores. Each string has one letter per population, with p indicating a pulse of migration from the respective source population, and x indicating no migration from that population. For example, the model pp_xp has two events; the first event, pp, has discrete contributions from populations 1 and 2, and the second event, xp, has a contribution only from population 2.

Optimization for each model was performed using a brute force search over a grid of parameter points, followed by a local refinement from the maximum likelihood grid-point. Segments below 11.7cM (corresponding to the first two bins in our histogram) were not used in the optimization process, as the number of short segments may be particularly affected by false positives. However, model predictions for these segments were reasonably accurate for all models and cohorts. In the likelihood optimization, the total ancestral proportions for the population were held fixed; the optimization was performed over the timing of the admixture events and the relative contributions of the distinct pulses of admixture from the same source. The resulting histories and corresponding likelihoods are shown in S8 Fig.

In addition to the global ancestry proportions, the pp model has a single free parameter (the timing of admixture), whereas the pp_xp model has three (two times of admixture and the relative contributions of the first and second non-African admixture). The pp_xp model outperforms the pp model by 631 log-likelihood units in the HRS, and by 839 log-likelihood units in the SCCS. We can reject the pp model according to either the Akaike information criterion or the Bayesian information criterion with *n* = 100 data points (one point per bin and per population).

#### Three-population admixture models

In the three-population case, the pxp_xpx model consists of a founding admixture of African and Native American migrants, followed by a subsequent pulse of European admixture. The ppp_xpx model consists of a founding admixture event involving the three populations, followed by a subsequent pulse of European admixture. The pxp_xpx_xpx model has a founding admixture of African and Native American ancestors, followed by two pulses of European admixture. Finally, the xpp_pxx_xpx model has a founding population of Europeans and Native Americans, followed by a pulse of African admixture, followed by a pulse of European admixture. These histories are shown in S9 Fig. The best-fit model for the SCCS and HRS is the pxp_xpx_xpx (see S10 and S9 Figs).

In addition to the three global ancestry proportions, model pxp_xpx, has two free time parameters. Model ppp_xpx has three parameters (two times of admixture and one relative contribution between the first and second pulse). Models xpp_pxx_xpx and pxp_xpx_xpx each have four parameters (three times and one relative contribution). For the HRS and SCCS datasets, the pxp_xpx_xpx model has the best likelihood. Since it outperforms the simpler models by 200 log-likelihood units, it is supported by either the Akaike information criterion or the Bayesian information criterion with *n* = 150 data points. Finally, to compare the effect of discrete-pulse models with that of continuous models, we performed inference with an additional pxp_xcx model, where xcx represents a period of constant European admixture. This model has three time parameters (one Native American and African admixture time, and the times of start and end of the European admixture).

#### Confidence intervals for timing estimates

Confidence intervals for all parameter values were obtained via bootstrap (S4 Table). For each model, we generated 100 bootstrap populations by resampling individuals with replacement. We performed parameter inference for each bootstrap population, and computed the 95% confidence interval of the resulting distribution of parameters. These confidence intervals account for the finite number of individuals in the sample. However, they do not account for biases resulting from population structure or model mis-specification. Because of the large sample size, these biases are likely more important than the uncertainty measured by the bootstrap.

## Supporting Information

S1 Text Supporting Material. (PDF)

S1 Fig Principal component analysis of all samples in the HRS, SCCS, and ASW cohorts, using SNPRelate [43]. The vertical axis (the first PC) corresponds to the distribution of African versus European component, whereas the horizontal axis indicates the distribution of Native American or Asian versus European component.
(TIF)

S2 Fig Global ancestry proportions of ASW, African-Americans in HRS, and SCCS individuals, calculated using the RFMix-inferred local ancestry. Blue, red, and yellow respectively denote African, European, and Native American or Asian ancestries. Each vertical line represent one individual, and the height of the color bars denoted the percentage of their respective ancestries in that individual.
(TIF)

S3 Fig Global ancestry proportions on the X chromosome for HRS African-American males (top) and females (bottom). Each vertical bar represents one individual. Blue, red, and yellow respectively denote African, European, and Native American or Asian ancestries.
(TIF)

S4 Fig Inferred Native American versus European ancestry in the SCCS cohort. (TIF)

S5 Fig Global ancestry estimates for all ASW individuals (top) and for individuals with more than 2% Native American ancestry in HRS (middle) and SCCS (bottom). Yellow, blue, red, and green represent, respectively, Native American, African, northern European, and southern European ancestries. Each column represents one individual. Individuals denoted by arrows in the middle plot are self-identified Hispanic African-Americans in HRS.
(TIF)

S6 Fig Inferred proportion of southern European ancestry within the total European ancestry versus that of Native American ancestry for African-Americans. Red represents self-identified Hispanic African-Americans in HRS, black represents SCCS African-Americans in Louisiana, and blue and green correspond, respectively, to other HRS and SCCS African-Americans.
(TIF)

S7 Fig Correlation between continental ancestry (African, European, and Native American/Asian) estimates from RFMix and ADMIXTURE for HRS African-American individuals. (TIF)

S8 Fig Estimated histories for two-population models, with the corresponding log-likelihoods. African ancestry is displayed in blue, and non-African ancestry in red. Rectangles show the proportion of each ancestry at each generation. Pie charts represent migrations, with the size of the pie representing the amounts of migrants at a given generation, and the sectors represent the proportion of migrants coming from each source population.
(TIF)

S9 Fig Estimated histories for three-population models, with the corresponding log-likelihoods. African ancestry is displayed in blue, European ancestry in red, and Native American ancestry in yellow. Rectangles show the proportion of each ancestry at each generation. Pie charts represent migrations, with the size of the pie representing the amounts of migrants at a given generation, and the sectors represent the proportion of migrants coming from each source population.
(TIF)

S10 Fig Comparison between observed tract length distribution (dots) and expectation under the best-fitting model (solid lines) for (a) the HRS and (b) the SCCS. Shaded areas represent one standard deviation departures from model expectations.
(TIF)

S11 Fig Estimated number of generations since admixture in HRS by region, assuming a single admixture pulse model in each region. (TIF)

S12 Fig IBD relatedness among African-Americans (top row) and among European-Americans (bottom row) across the US census regions (using 2010 region of residence). In each subfigure, the thickness and opacity of the line connecting any two regions show the strength of relatedness between those regions. Note that scaling of lines is not equal across different subfigures, and relatedness between regions with fewer than 10,000 possible pairs of individuals is not shown (see Materials and Methods for details).
(TIF)

S13 Fig IBD relatedness among African-Americans (top row) and among European-Americans (bottom row) across the US census regions (using the regions of birth). In each subfigure, the thickness and opacity of the line connecting any two regions show the strength of relatedness between those regions. Note that scaling of lines is not equal across different subfigures, and relatedness between regions with fewer than 10,000 possible pairs of individuals is not shown (see Materials and Methods for details).
(TIF)

S14 Fig Relatedness between African-Americans across US census regions based on the average total length of shared IDB segments of length in the specified ranges (using region of residence in 2010). The values shown in the second row are converted to grayscale in the top row to aid visualization, with the scales presented underneath each figure. Since the matrices are symmetric, only the upper-triangular parts are shown.
(TIF)

S15 Fig Relatedness between European-Americans across US census regions based on the average total length of shared IDB segments of length in the specified ranges (using region of residence in 2010). The values shown in the second row are converted to grayscale in the top row to aid visualization, with the scales presented underneath each figure. Since the matrices are symmetric, only the upper-triangular parts are shown.
(TIF)

S16 Fig Relatedness between African-Americans and European-Americans across US census regions based on the average total length (top and middle rows) and number (bottom row) for IDB segments of length in the specified ranges (using region of residence in 2010). The values shown in the second row are converted to grayscale in the top row to aid visualization, with the scales presented underneath each figure. The columns in each figure represent European-Americans, and the rows represent African-Americans.
(TIF)

S17 FigCensus-based predicted relatedness between (a) African-Americans and (b) European-Americans across the US census regions.The top row shows the directional metric $\bar{P}$, whereas the bottom row shows the symmetric one *I*. In the top figures (read column-wise), each column shows for its respective census region the proportion of ancestral population which originated from other census regions. See S18 Fig for the numerical values of these regional relatedness metrics.(TIF)

S18 FigCensus-based predicted relatedness between (a) African-Americans and (b) European-Americans across the US census regions.The top row shows the values for the directional metric $\bar{P}$, whereas the bottom row shows those for the symmetric one *I*. In the top figures (read column-wise), each column shows for its respective census region the proportion of ancestral population which originated from other census regions.(TIF)

S19 Fig Decay of IBD sharing with distance, calculated for the SCCS cohort, for IBD segments of length 3cM or longer (top), 10cM or longer (middle), and 18cM or longer (bottom). The plot is in log-linear scale, and the dashed lines represent two standard error deviations from the mean for the corresponding curve.
(TIF)

S20 Fig Estimated decay of IBD sharing with distance for IBD segments of length 3cM or longer (top), 10cM or longer (middle), and 18cM or longer (bottom). Points represent the data and lines represent the model.
(TIF)

S21 Fig Distribution of IBD sharing for African-American (blue) and European-American (red) individuals using IBD tracts belonging to different length bins. (TIF)

S22 Fig Number of non-Hispanic US-born HRS individuals moving from one region to the other between their time of birth and the 2010 sampling year. Rows represent regions of birth, and columns represent regions of residence in 2010.
(TIF)

S1 Table Characteristics of African-Americans in the HRS and SCCS cohorts. (PDF)

S2 Table Number of US-born non-Hispanic individuals (HRS, SCCS, and ASW combined) by race and census region or division of residence in 2010 (color coded to match the IBD maps shown in the main text). (PDF)

S3 TableDistribution of birth years in HRS African-Americans.Confidence intervals for selected models inferred using Tracts. Here, *t*_*i*_ refers to the time of the *i*th migration event (in generations ago), and $f_{2}^{\text{EUR}}$ refers to the fraction of European admixture in the second migration event.(PDF)

S4 TableConfidence intervals for selected models inferred using Tracts.Here, *t*_*i*_ refers to the time of the *i*th migration event (in generations ago), and $f_{2}^{\text{EUR}}$ refers to the fraction of European admixture in the second migration event.(PDF)

S5 Table Related pairs of individuals in the cohorts with estimated kinship coefficient of 0.1 or larger. For relateds within HRS, we used the list provided by the Health and Retirement Study.
(PDF)
