# Supplementary material for: The Great Migration and African-American Genomic Diversity
Source: PLoS Genet. 2016 May 27;12(5):e1006059. doi: 10.1371/journal.pgen.1006059 (PMC4883799; doi:10.1371/journal.pgen.1006059)
Supplement: S1 Text — (PDF) [file pgen.1006059.s001.pdf]

Supporting Material

The Great Migration and African-American genomic diversity

Soheil Baharian, Maxime Barakatt, Christopher R. Gignoux, Suyash Shringarpure, Jacob Errington, William J. Blot, Carlos D. Bustamante, Eimear E. Kenny, Scott M. Williams, Melinda C. Aldrich, Simon Gravel

Contents

1 Principal component analysis **1**

2 Detailed geographic information on HRS and SCCS cohorts **1**

3 List of related individuals **2**

4 Distribution of shared IBD tracts **2**

5 Regional IBD relatedness and sampling locations **3**

1 Principal component analysis

The results of a PCA analysis on the combined HRS, SCCS, and ASW are shown in S1 Fig.

The ASW and SCCS samples cluster with the African-American samples in HRS, as expected. The vertical axis shows that African ancestry in African-Americans varies continuously in all cohorts. African-Americans with Hispanic ethnicity are positioned slightly away from (towards the right of) the cluster of non-Hispanic African-Americans, in the same direction as other non-African-American Hispanic individuals and along the axis corresponding to Native American or Asian component. Interestingly, there are individuals in the ASW cohort with very high levels of Native American or Asian component. Specifically, 1 ASW sample lies almost halfway between the European and the Asian cluster, with almost no African component present, and 4 ASW samples have very high proportions of Native American or Asian component. Similarly, within HRS, 5 African-American samples – who have *not* self-identified as Hispanics – have very high proportions of Native American or Asian component with 4 of them having extremely low African component. Analogous to these 4 samples, there is one African-American sample – who has self-identified as Hispanic – who has a similar pattern of African, European, and Native American or Asian ancestry.

2 Detailed geographic information on HRS and SCCS cohorts

The detailed geographic information of individuals in HRS and SCCS is used in our downstream analyses in the following manner.

HRS. The restricted HRS data contains ZIP codes for each individual, but not states. To calculate per-state global ancestry proportions within HRS, we use the following commands in Mathematica (version 10.1.0) to get the list of ZIP codes within each state in the contiguous US.

```
states = CountryData["UnitedStates", "Regions"];
states = Delete[states, {Position[states, "Alaska"][[1]], Position[states, "Hawaii"][[1]]}];
ZIPcodes = GeoEntities[Entity["USState", #], "ZIPCode"][[All, 2]]& /@ states;
```

For each state, we select HRS individuals whose ZIP codes belong to that state, then estimate the mean ancestry proportions for the state using the selected individuals. To find the spatial distance between HRS individuals, we use the ZIP Code Tabulation Area (ZCTA) database (from the 2014 US Gazetteer Files by the US Census Bureau, at `https://www.census.gov/geo/maps-data/data/gazetteer2014.html`) to assign latitude and longitude coordinates to the individuals based on their ZIP codes. Each coordinate in the ZCTA database is essentially the latitude and longitude coordinates of the geographic centroid of the corresponding ZIP Code Tabulation Area, as defined by the US Census Bureau. We then calculate the geodesic distance between a pair of individuals given their assigned geographic coordinates, as an estimate for the actual distance between the two individuals.

SCCS. The data we received from SCCS contains latitude and longitude coordinates of clinics participating in the study. To convert the coordinates into specific locations (e.g., ZIP codes, states, and census regions), we used the Nominatim service from OpenStreetMap to perform reverse geocoding of the coordinates. (Data available under the Open Database Licence; © OpenStreetMap contributors.) Specifically, we used the OpenStreetMap API provided through MapQuest due to its unlimited usage policy (details at `http://open.mapquestapi.com/nominatim/#reverse`).

3 List of related individuals

We have found the pairs of individuals denoted in S5 Table to have kinship coefficients of 0.1 or greater, as estimated by PLINK. To be consistent with the definition from HRS, we have therefore labeled these pairs as related individuals and have excluded their contributions from our IBD analyses (see Materials and Methods).

4 Distribution of shared IBD tracts

For each individual in the HRS, SCCS, and ASW cohorts, we calculate the number of IBD segments that he or she shares with *all other* non-related individuals across all cohorts, using the sparse relatedness matrix $\mathcal{N}$ calculated above (which contains the total *number* of shared IBD segments between each pair of individuals). This distribution of IBD sharing for segments in different length bins is shown in S21 Fig. For short segments, Europeans show substantially more IBD compared to African-Americans, and there is more variation in the number of IBD tracts. This is due to differences in sample size and historical effective population size. The difference in variance could reflect the greater contributions of more recent migrants in European-Americans or, more generally, the presence of population structure that persisted throughout the US history. By contrast, African-Americans, on average, have more long IBD. Since long segments represent recent history, we attribute this difference between short and long segments to a much greater reduction in effective population size in African-Americans compared to European-Americans since the arrival into the Americas.

IBD sharing for European-Americans tends to be mostly via shorter (and, therefore, older) IBD segments compared to that for African-Americans. Hence, for bins containing longer IBD segments, the peak corresponding to European-American IBD sharing moves to the left faster compared to the respective peak for African-Americans.

5 Regional IBD relatedness and sampling locations

Movement of HRS individuals between their time of birth and the 2010 sampling year is represented in S22 Fig. Because of these migrations, the total number of individuals in each census region or division depends on whether we assign each individual to their region/division of birth or region/division of residence in 2010. We chose the latter assignment criterion throughout our analyses, unless otherwise mentioned.

Using the individuals' region of residence in 2010 as their location, we find the relatedness pattern shown in S12 Fig between census regions for African-Americans and European-Americans. On the other hand, using the individuals' region of birth as their location instead of their 2010 region of residence, we find the relatedness pattern shown in S13 Fig between census regions for African-Americans and European-Americans.

The differences between S12 Fig and S13 Fig are due to the following factors: If we use the 2010 region of residence as opposed to the region of birth as the individuals' locations, the number of individuals in the northern and western census regions increases due to the migrations from the South. Since we only visualize relatedness when at least 10,000 pairs of individuals exist between two regions, the number of connections shown differ between the two figures.

Moreover, we also see that the connection between West South Central and West North Central is weaker S12 Fig than in S13 Fig. This is mainly due to 21 African-American individuals in HRS who were born in West South Central and who have much IBD with other individuals who were born in northern regions, especially those in West North Central. These individuals later moved to West North Central and, thus, are sampled in the latter region in 2010 (see S22 Fig). S14 Fig shows details of the relatedness patterns among African-Americans across US census regions. The grayscale plots in the top row show the average pairwise IBD length shared between census regions, calculated using all IBD segments satisfying the length criteria shown below each column. Actual values are shown in the middle row, whereas plots in the bottom row show the total number of IBD segments shared between the US census regions. Relatedness between European-Americans across US census regions is similarly displayed in S15 Fig. Relatedness between African-Americans and European-Americans is shown in S16 Fig.
