# Supplementary figures and images for: The Great Migration and African-American Genomic Diversity
Source: PLoS Genet. 2016 May 27;12(5):e1006059. doi: 10.1371/journal.pgen.1006059 (PMC4883799; doi:10.1371/journal.pgen.1006059)

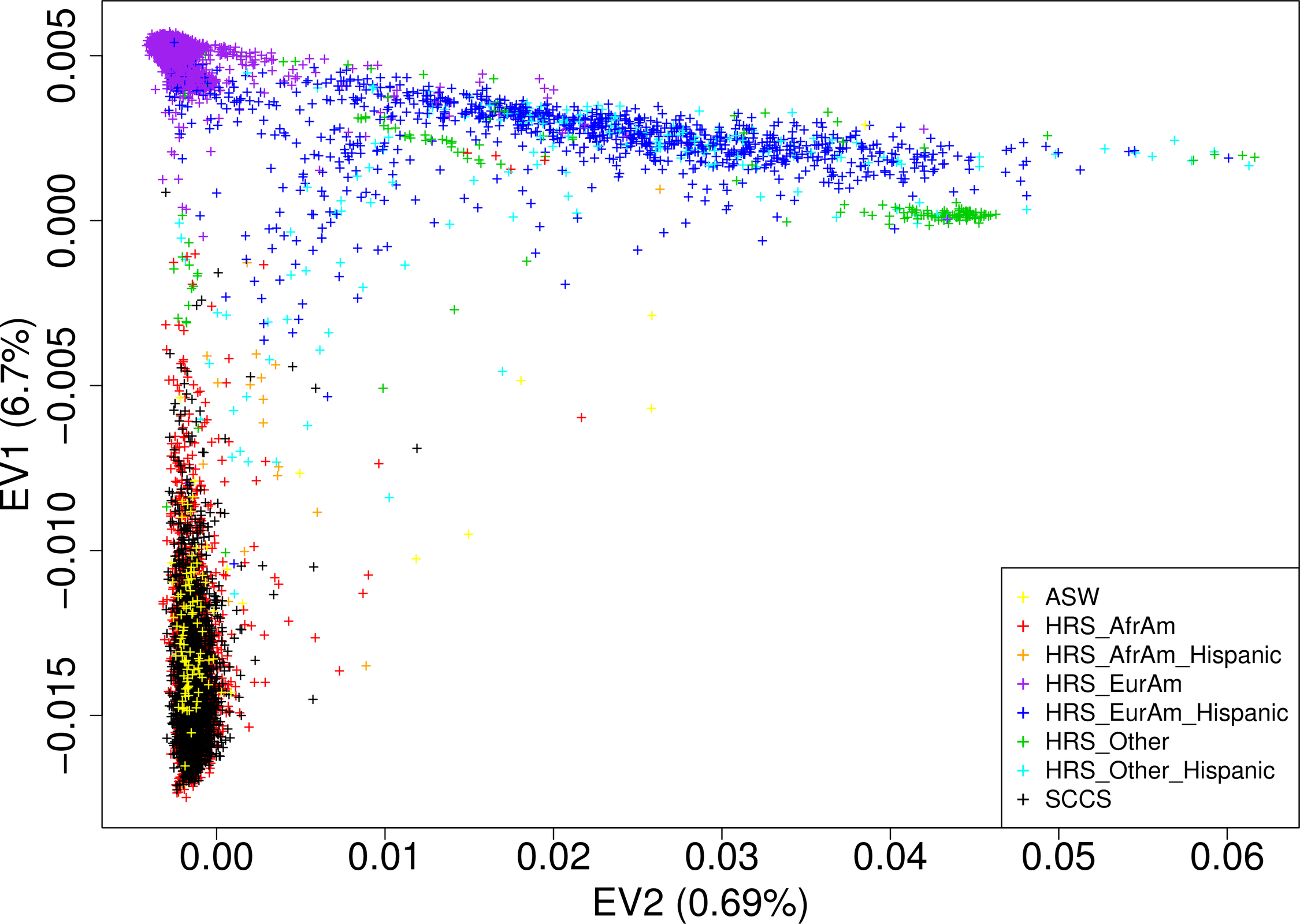

Supplement: S1 Fig — The vertical axis (the first PC) corresponds to the distribution of African versus European component, whereas the horizontal axis indicates the distribution of Native American or Asian versus European component. (TIF) [file pgen.1006059.s002.tif]

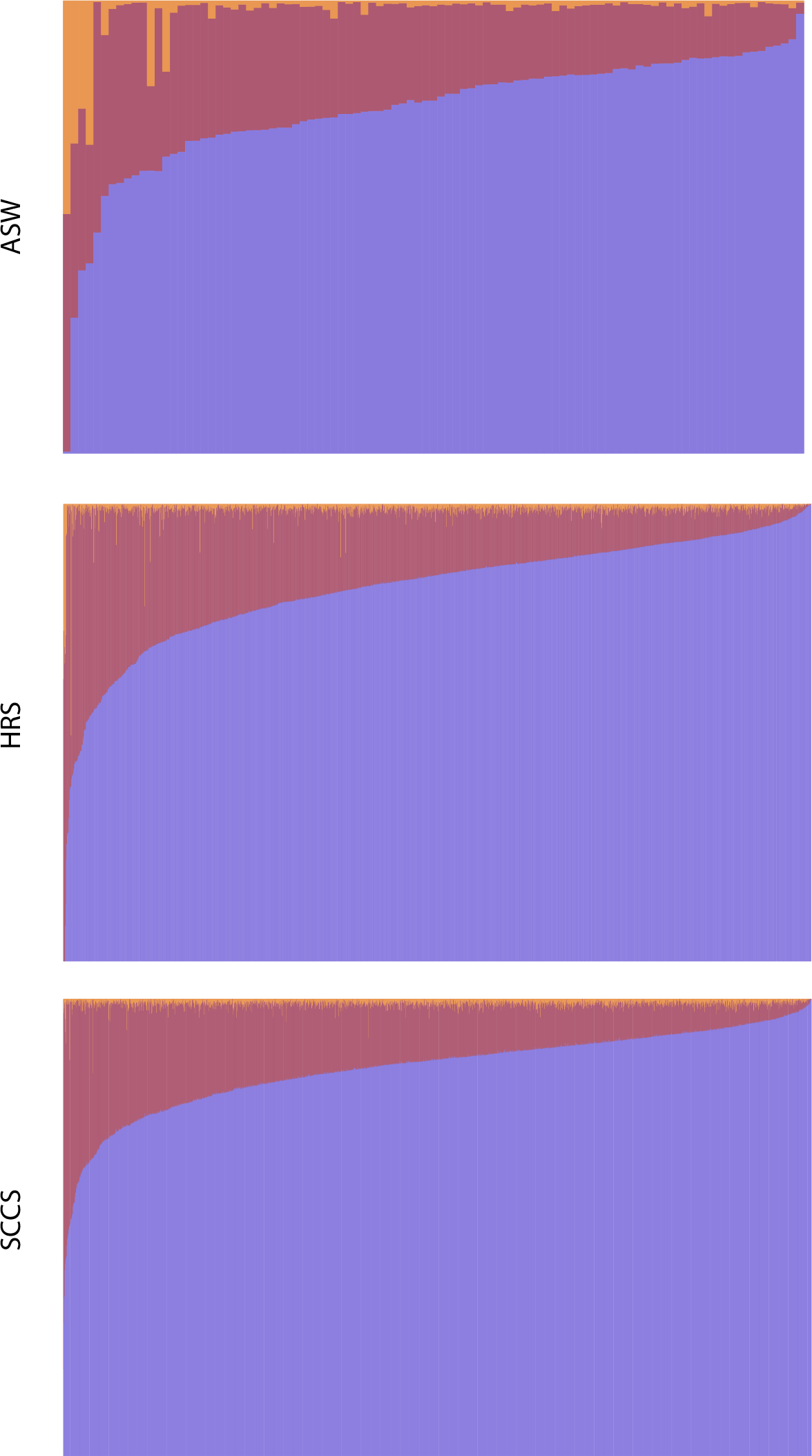

Supplement: S2 Fig — Blue, red, and yellow respectively denote African, European, and Native American or Asian ancestries. Each vertical line represent one individual, and the height of the color bars denoted the percentage of their respective ancestries in that individual. (TIF) [file pgen.1006059.s003.tif]

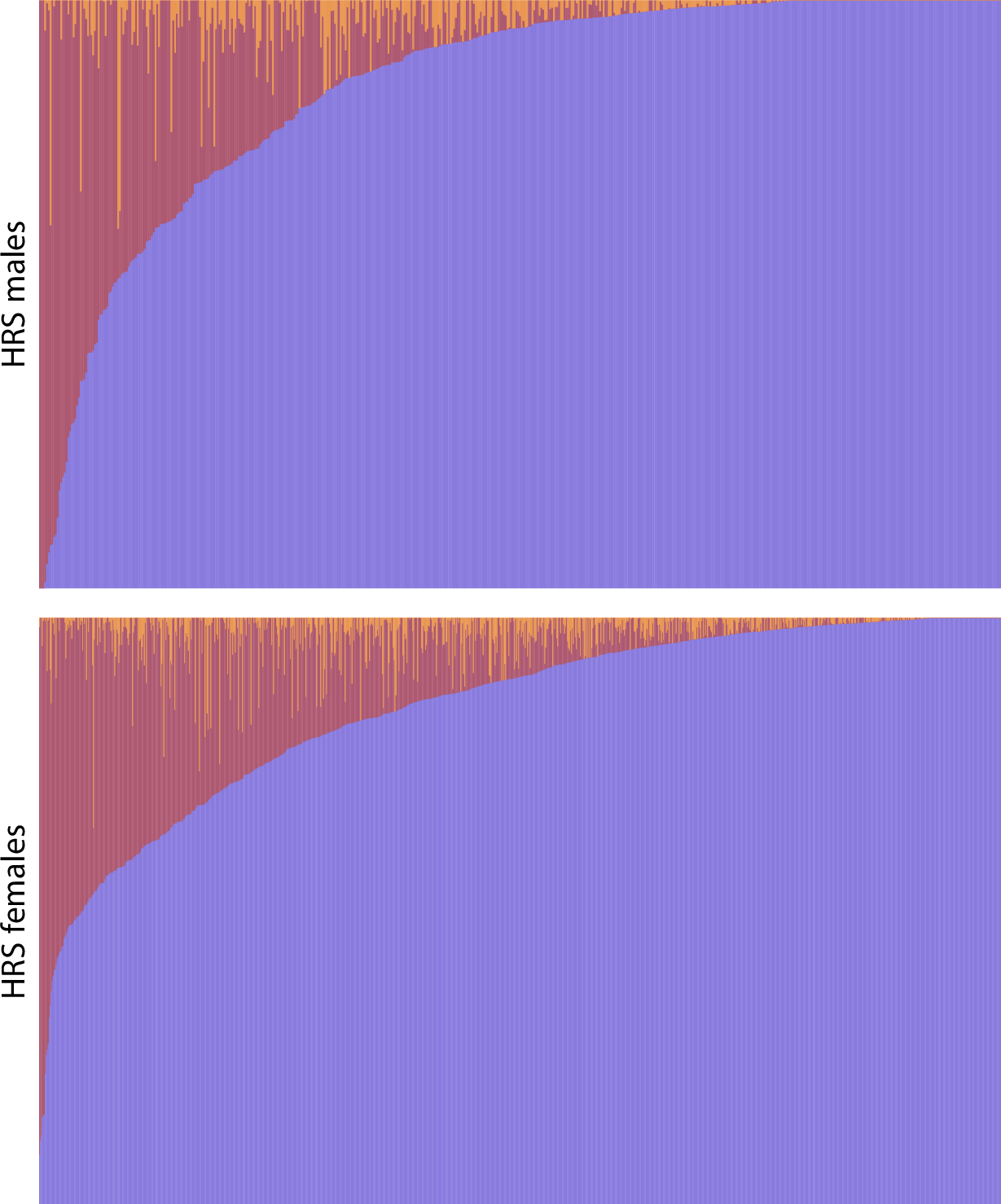

Supplement: S3 Fig — Each vertical bar represents one individual. Blue, red, and yellow respectively denote African, European, and Native American or Asian ancestries. (TIF) [file pgen.1006059.s004.tif]

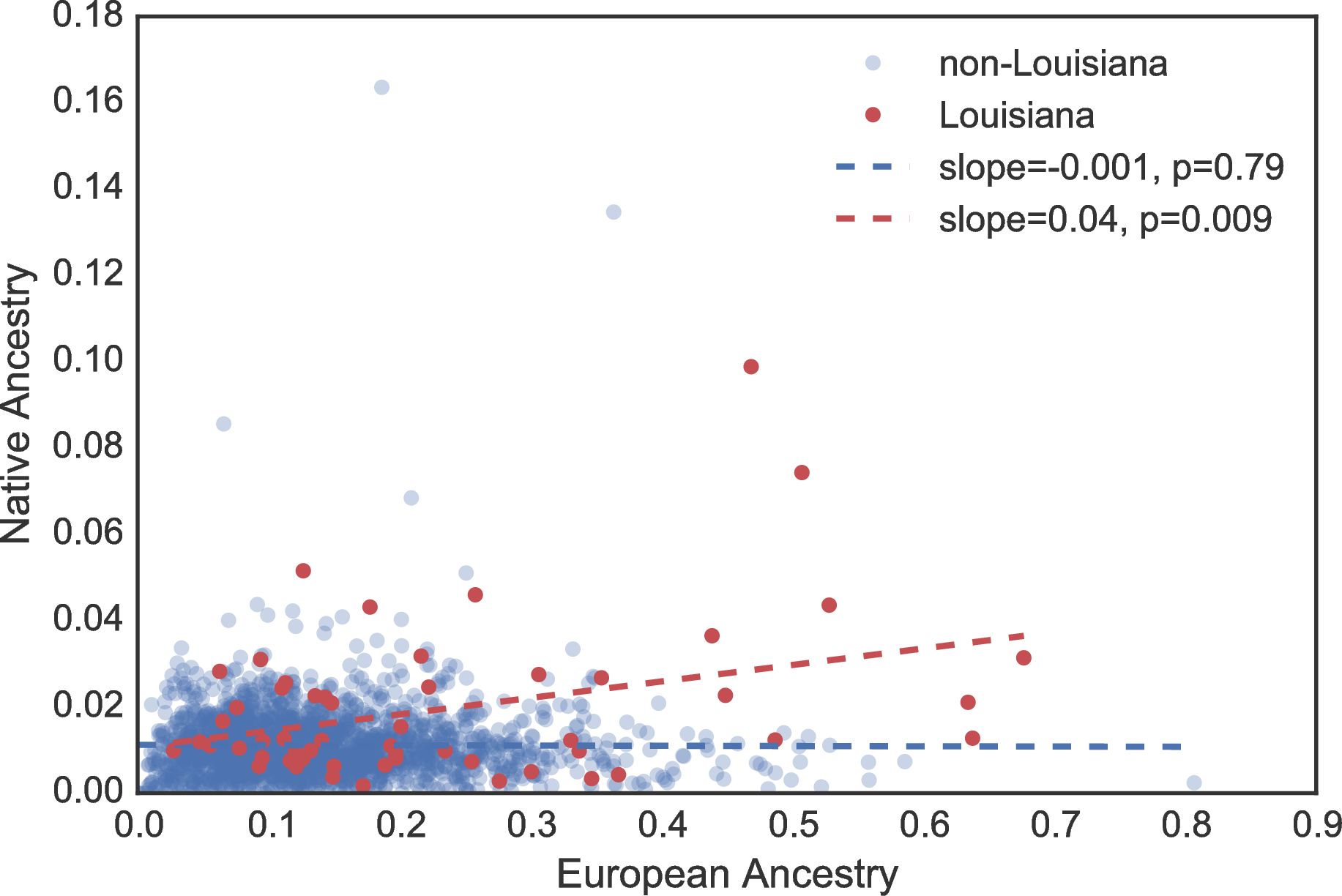

Supplement: S4 Fig — (TIF) [file pgen.1006059.s005.tif]

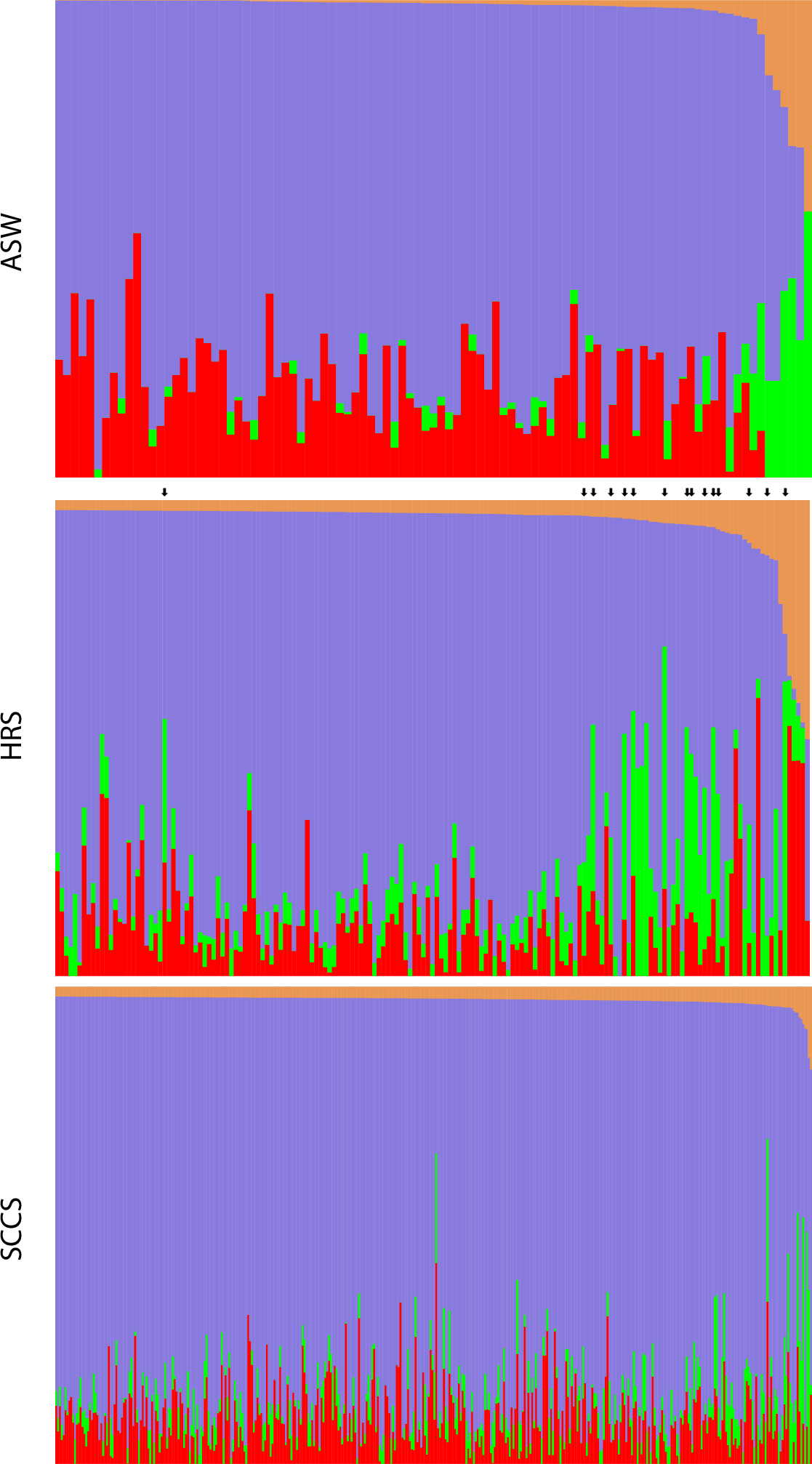

Supplement: S5 Fig — Yellow, blue, red, and green represent, respectively, Native American, African, northern European, and southern European ancestries. Each column represents one individual. Individuals denoted by arrows in the middle plot are self-identified Hispanic African-Americans in HRS. (TIF) [file pgen.1006059.s006.tif]

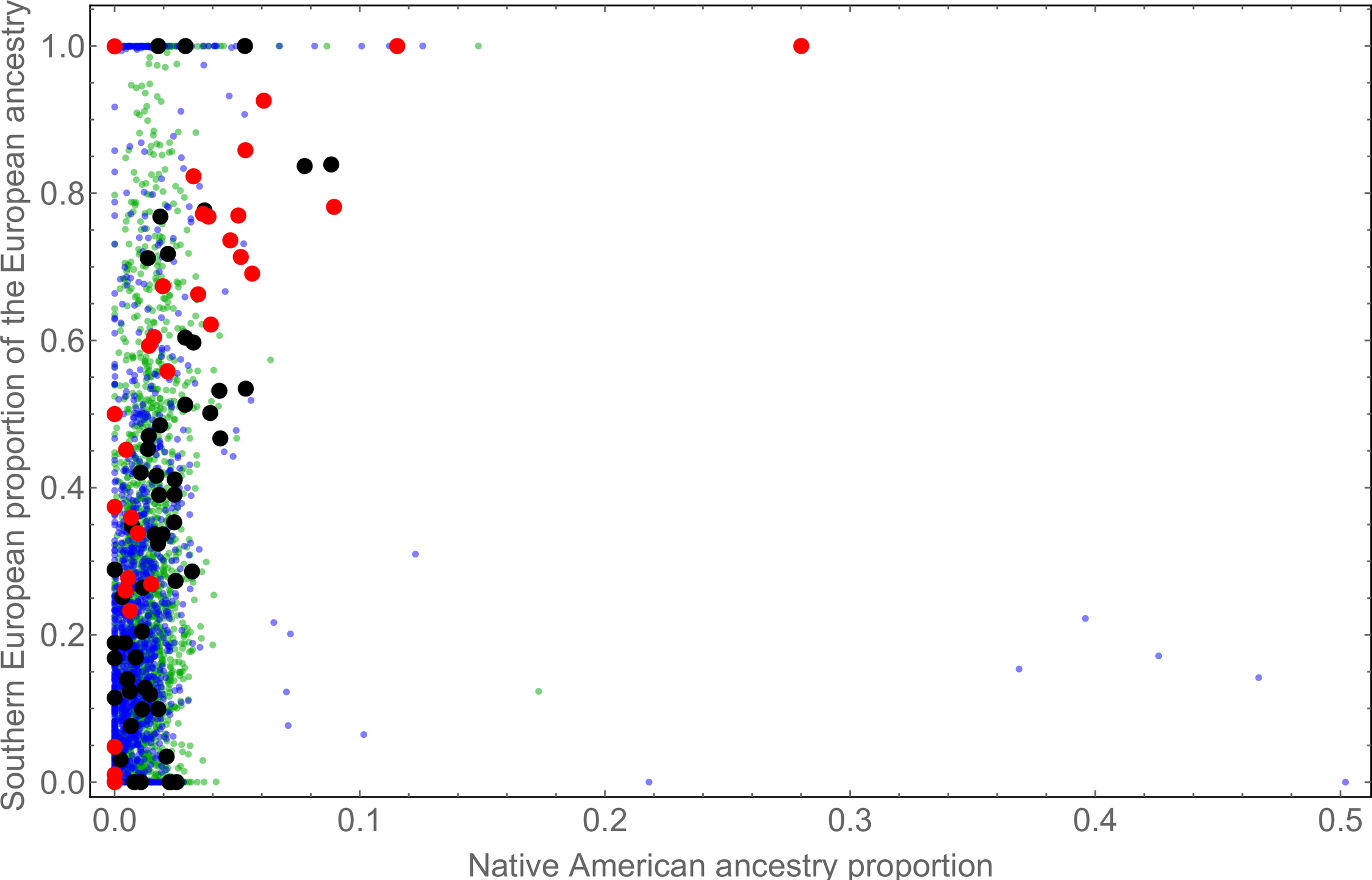

Supplement: S6 Fig — Red represents self-identified Hispanic African-Americans in HRS, black represents SCCS African-Americans in Louisiana, and blue and green correspond, respectively, to other HRS and SCCS African-Americans. (TIF) [file pgen.1006059.s007.tif]

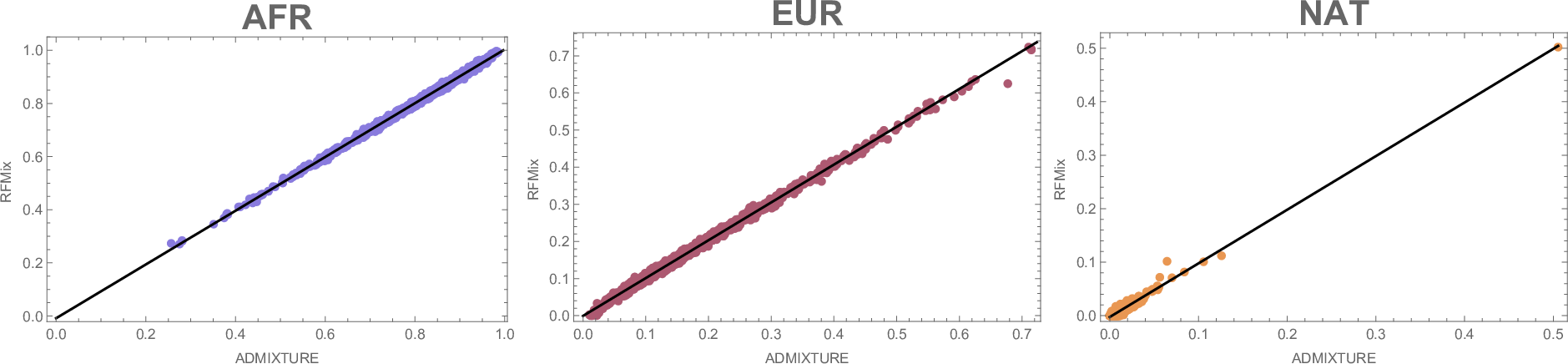

Supplement: S7 Fig — (TIF) [file pgen.1006059.s008.tif]

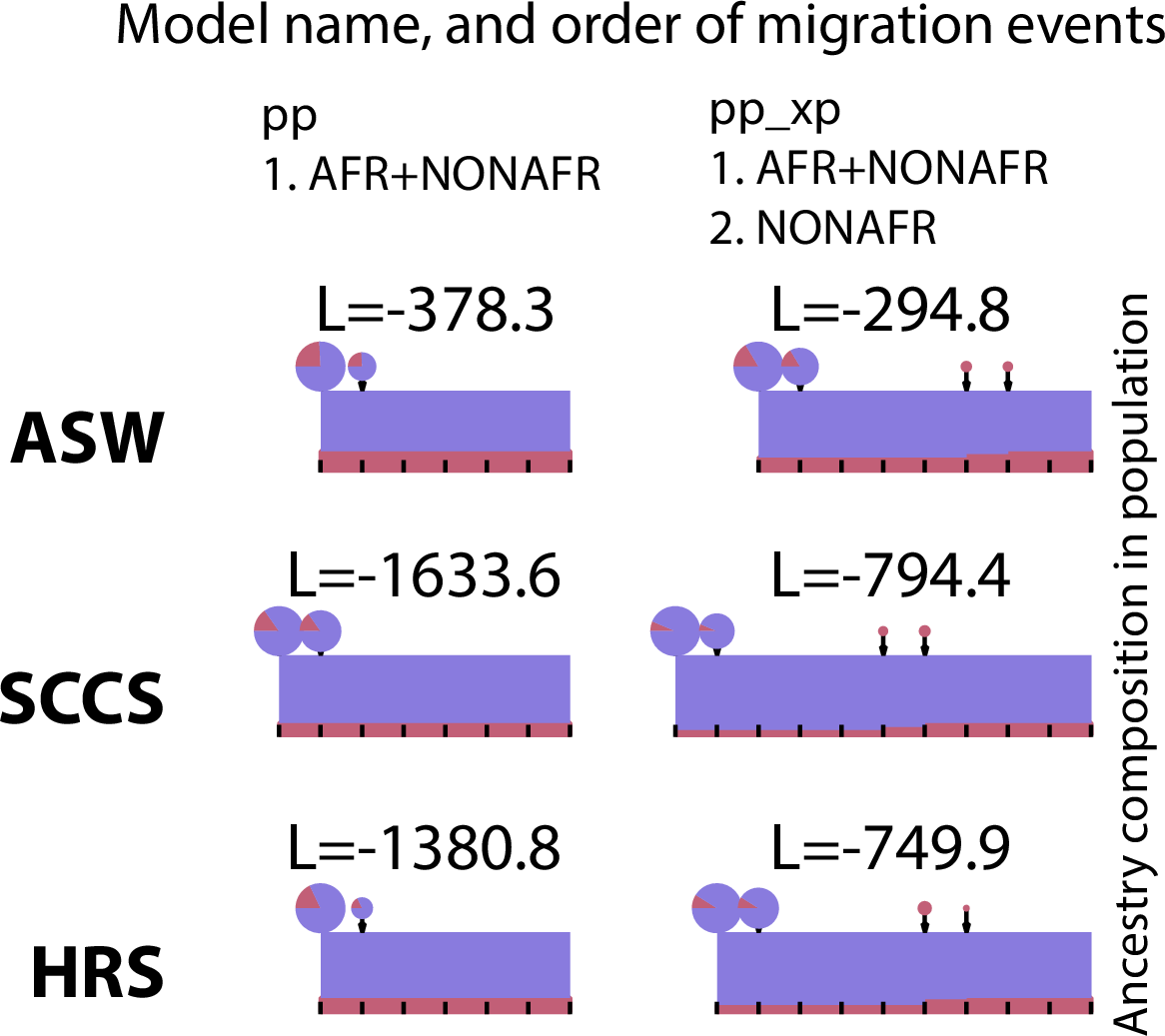

Supplement: S8 Fig — African ancestry is displayed in blue, and non-African ancestry in red. Rectangles show the proportion of each ancestry at each generation. Pie charts represent migrations, with the size of the pie representing the amounts of migrants at a given generation, and the sectors represent the proportion of migrants coming from each source population. (TIF) [file pgen.1006059.s009.tif]

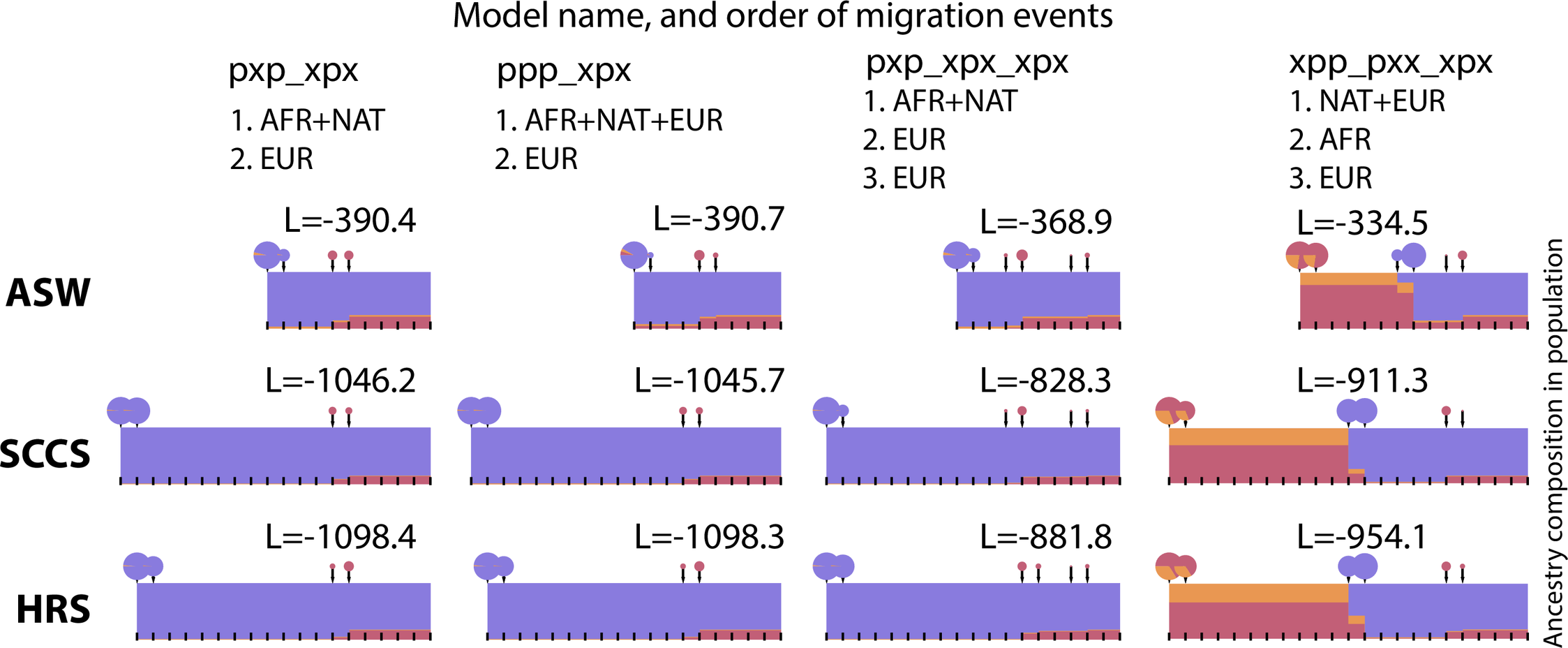

Supplement: S9 Fig — African ancestry is displayed in blue, European ancestry in red, and Native American ancestry in yellow. Rectangles show the proportion of each ancestry at each generation. Pie charts represent migrations, with the size of the pie representing the amounts of migrants at a given generation, and the sectors represent the proportion of migrants coming from each source population. (TIF) [file pgen.1006059.s010.tif]

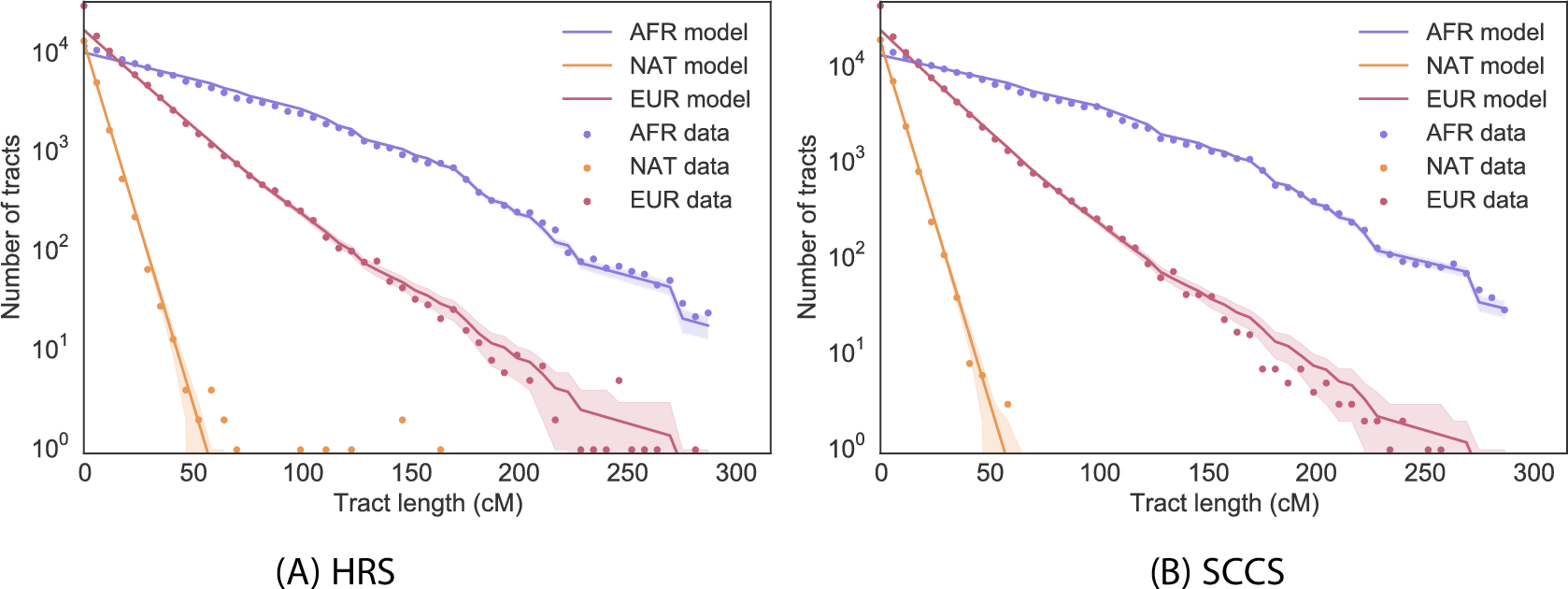

Supplement: S10 Fig — Shaded areas represent one standard deviation departures from model expectations. (TIF) [file pgen.1006059.s011.tif]

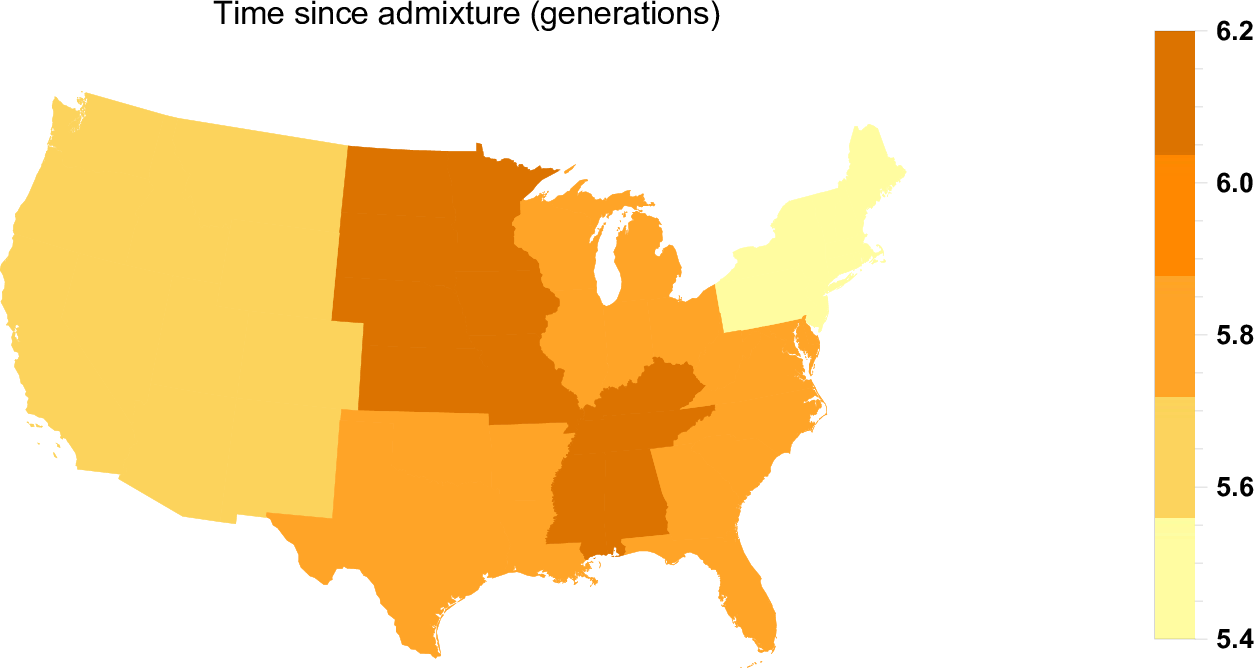

Supplement: S11 Fig — (TIF) [file pgen.1006059.s012.tif]

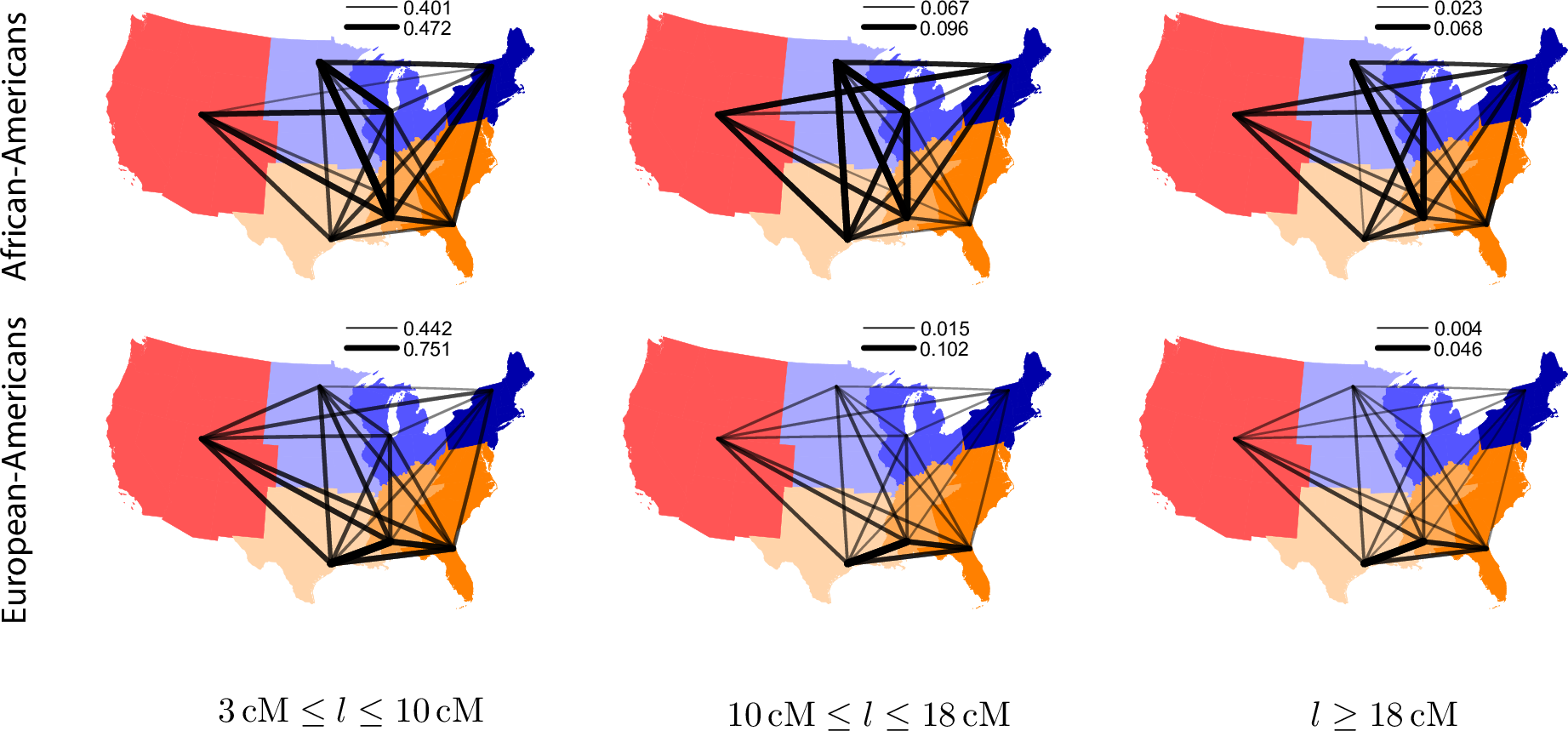

Supplement: S12 Fig — In each subfigure, the thickness and opacity of the line connecting any two regions show the strength of relatedness between those regions. Note that scaling of lines is not equal across different subfigures, and relatedness between regions with fewer than 10,000 possible pairs of individuals is not shown (see Materials and Methods for details). (TIF) [file pgen.1006059.s013.tif]

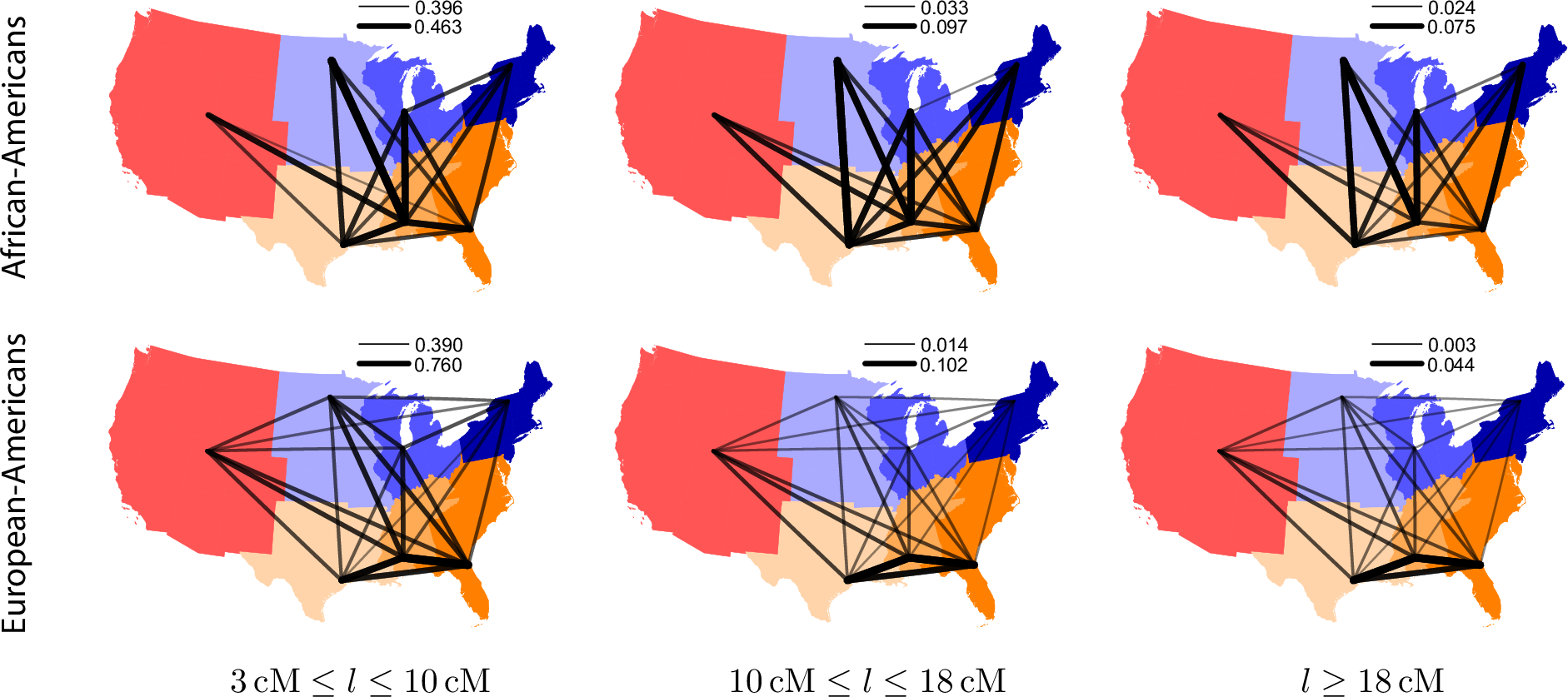

Supplement: S13 Fig — In each subfigure, the thickness and opacity of the line connecting any two regions show the strength of relatedness between those regions. Note that scaling of lines is not equal across different subfigures, and relatedness between regions with fewer than 10,000 possible pairs of individuals is not shown (see Materials and Methods for details). (TIF) [file pgen.1006059.s014.tif]

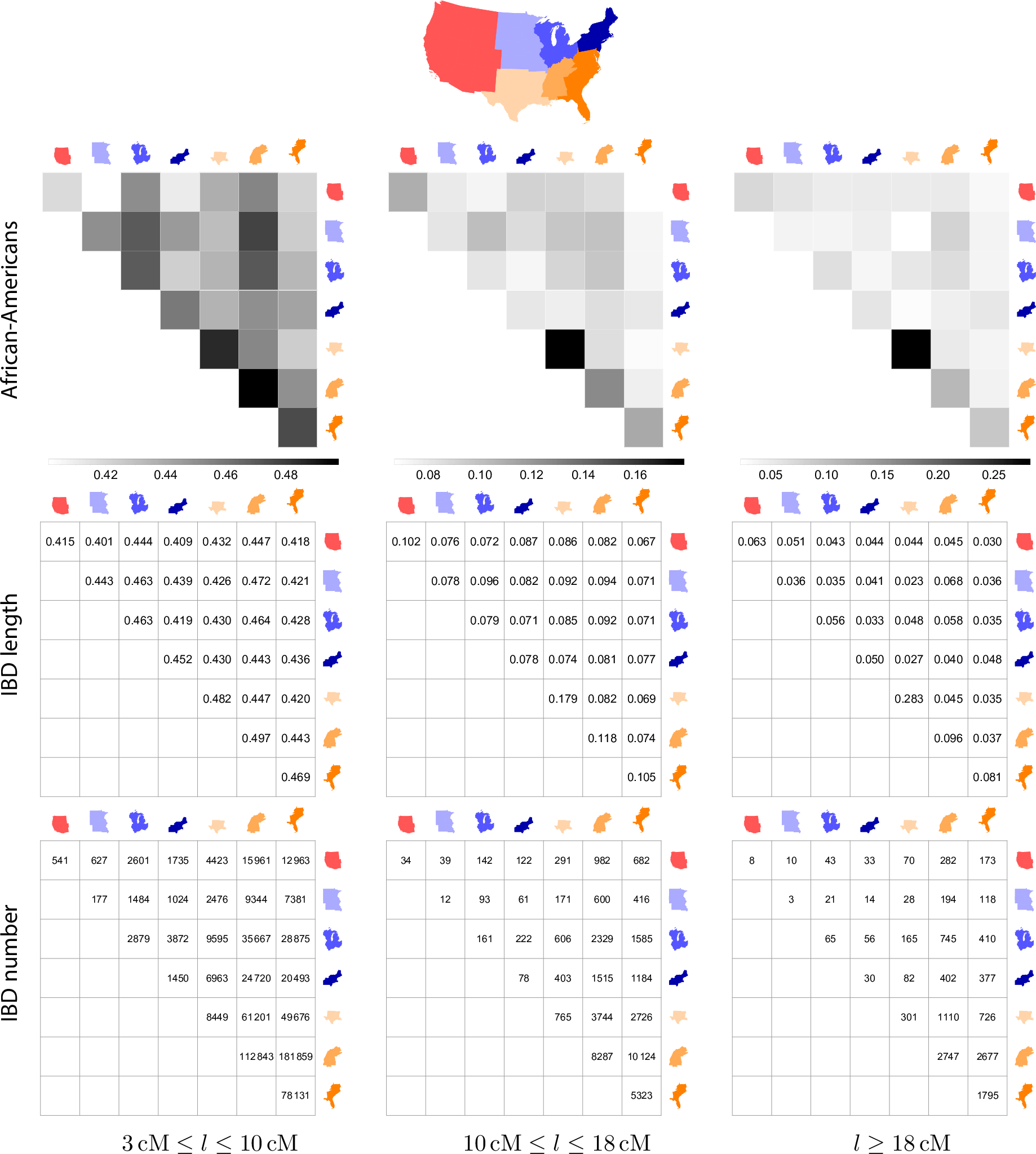

Supplement: S14 Fig — The values shown in the second row are converted to grayscale in the top row to aid visualization, with the scales presented underneath each figure. Since the matrices are symmetric, only the upper-triangular parts are shown. (TIF) [file pgen.1006059.s015.tif]

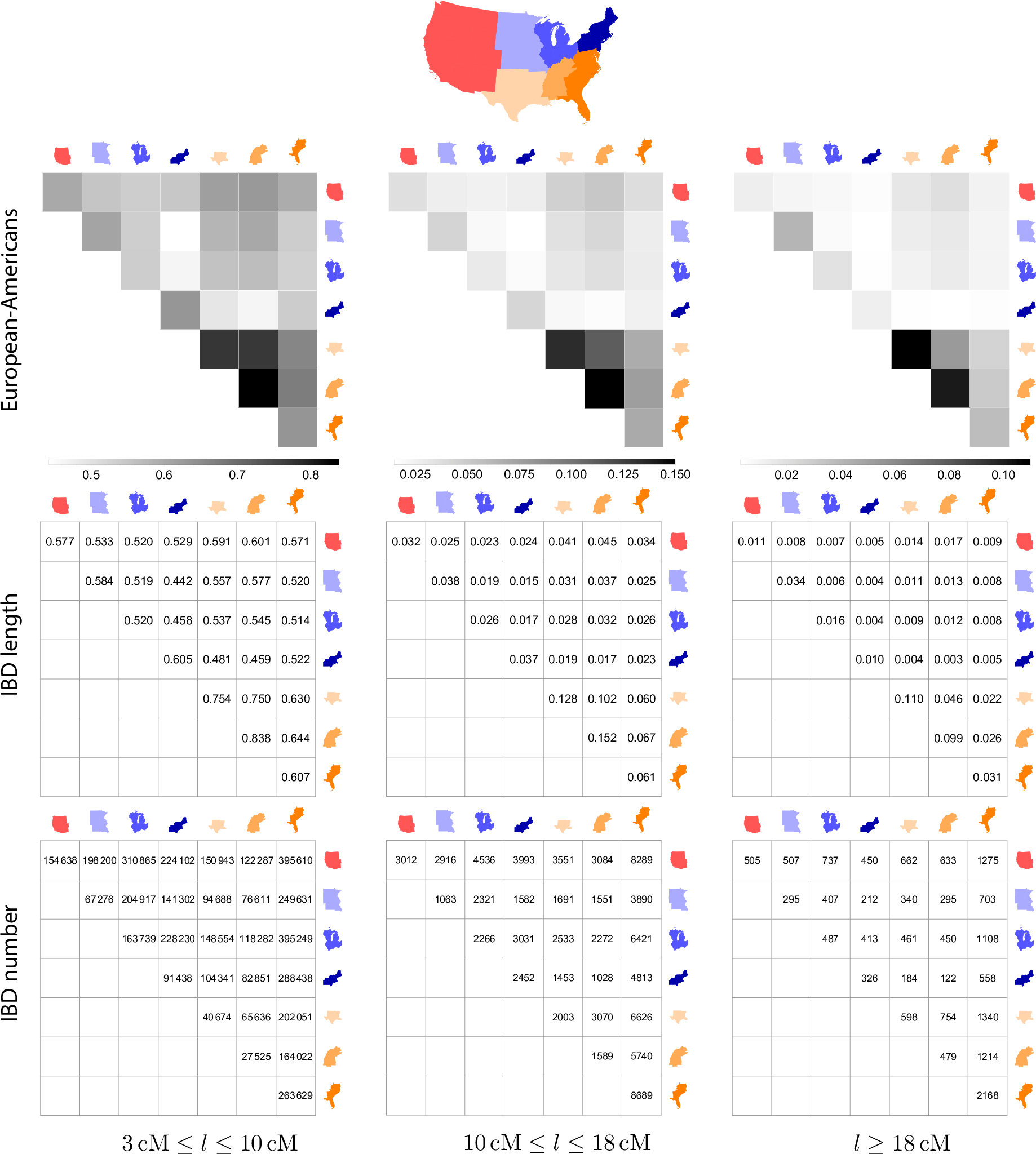

Supplement: S15 Fig — The values shown in the second row are converted to grayscale in the top row to aid visualization, with the scales presented underneath each figure. Since the matrices are symmetric, only the upper-triangular parts are shown. (TIF) [file pgen.1006059.s016.tif]

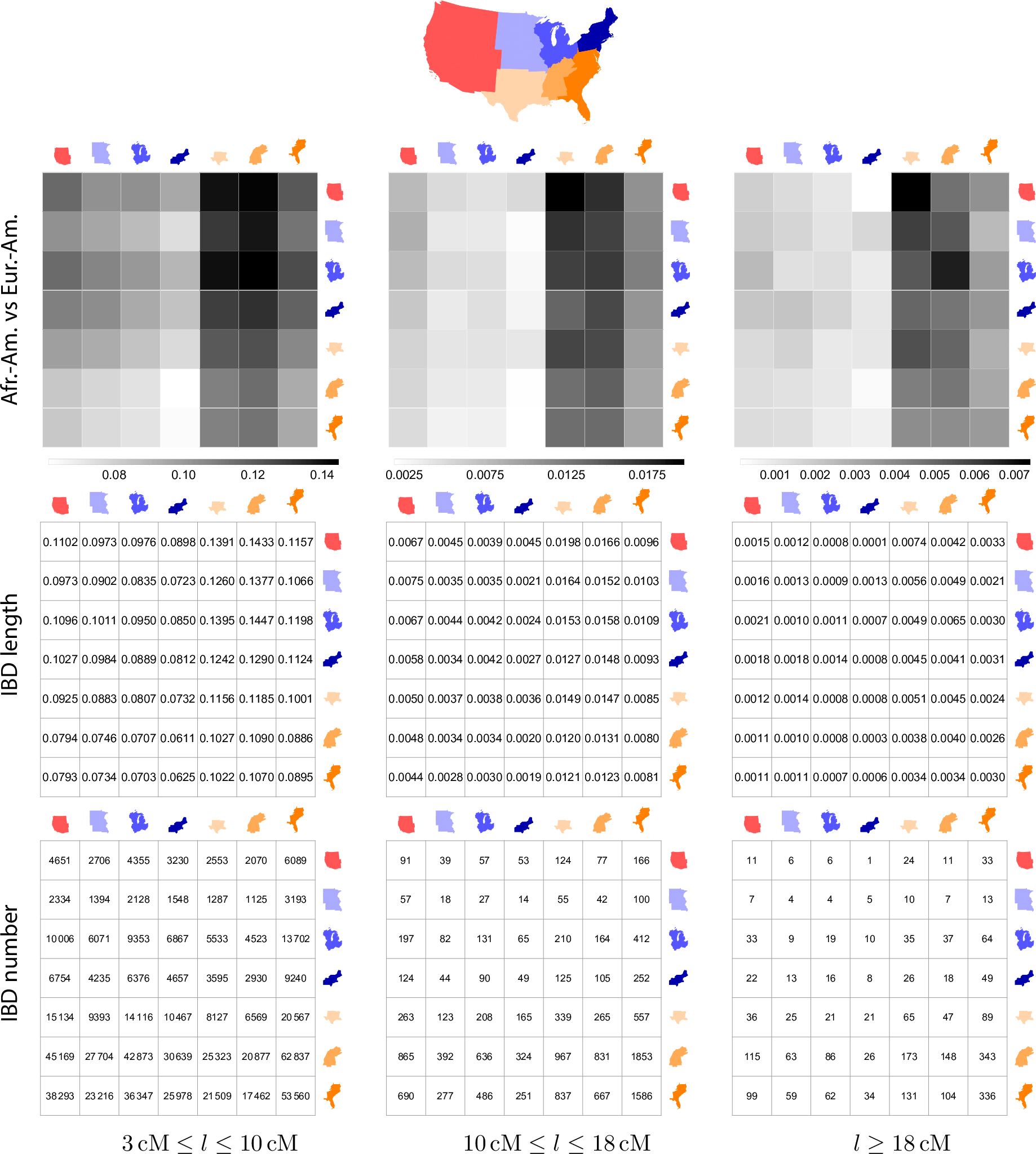

Supplement: S16 Fig — The values shown in the second row are converted to grayscale in the top row to aid visualization, with the scales presented underneath each figure. The columns in each figure represent European-Americans, and the rows represent African-Americans. (TIF) [file pgen.1006059.s017.tif]

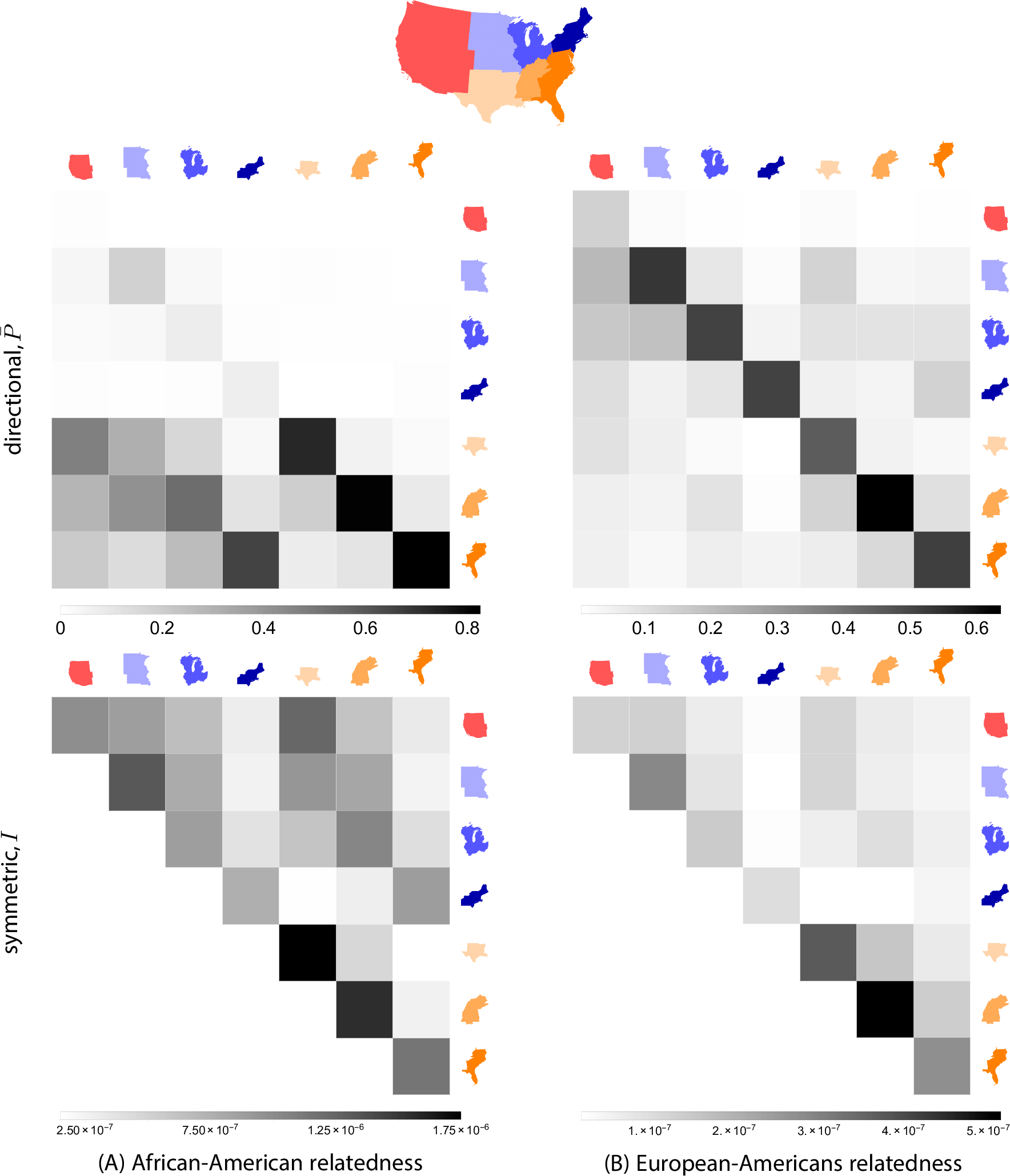

Supplement: S17 Fig — The top row shows the directional metric P‾, whereas the bottom row shows the symmetric one I. In the top figures (read column-wise), each column shows for its respective census region the proportion of ancestral population which originated from other census regions. See S18 Fig for the numerical values of these regional relatedness metrics. (TIF) [file pgen.1006059.s018.tif]

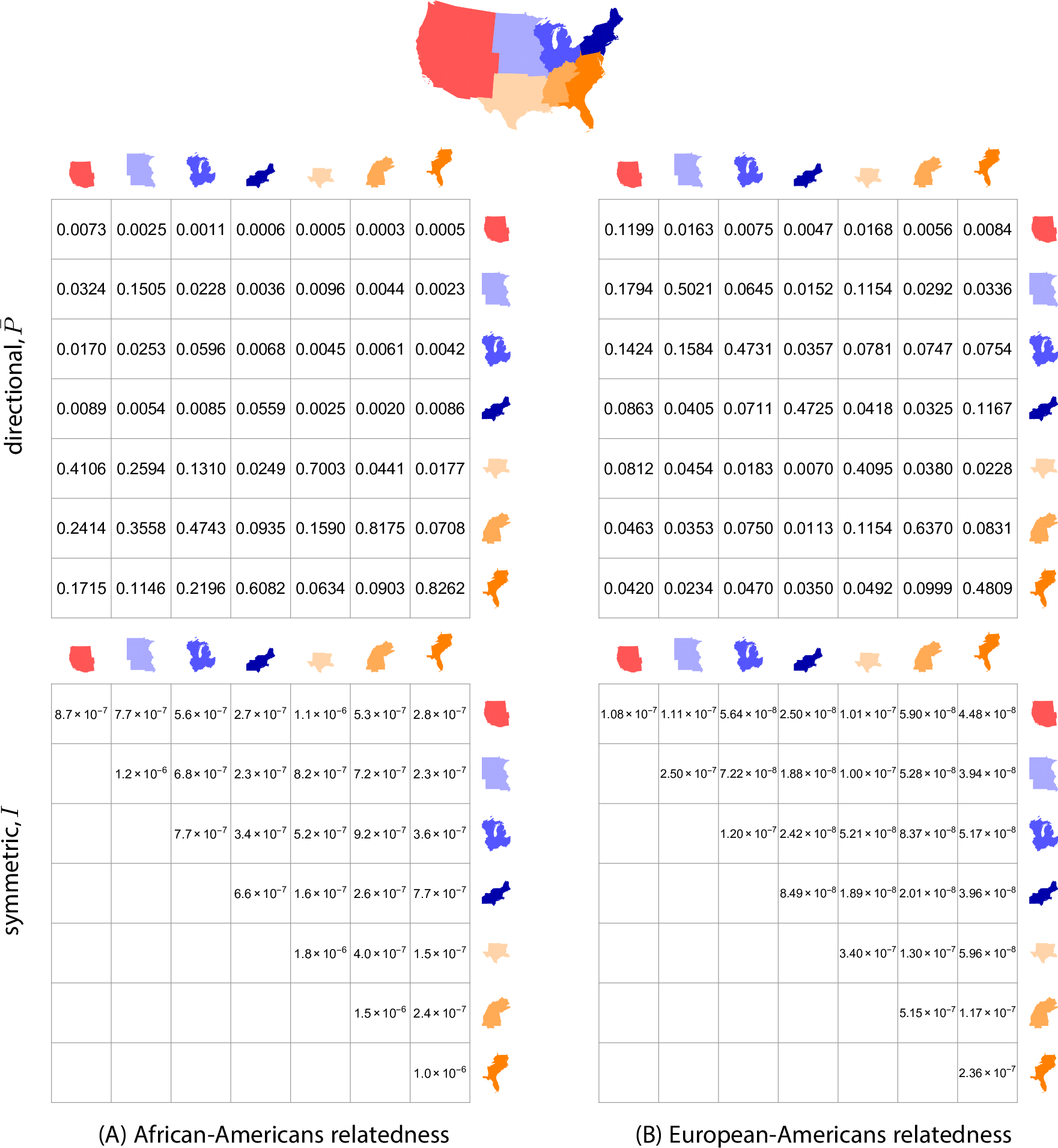

Supplement: S18 Fig — The top row shows the values for the directional metric P‾, whereas the bottom row shows those for the symmetric one I. In the top figures (read column-wise), each column shows for its respective census region the proportion of ancestral population which originated from other census regions. (TIF) [file pgen.1006059.s019.tif]

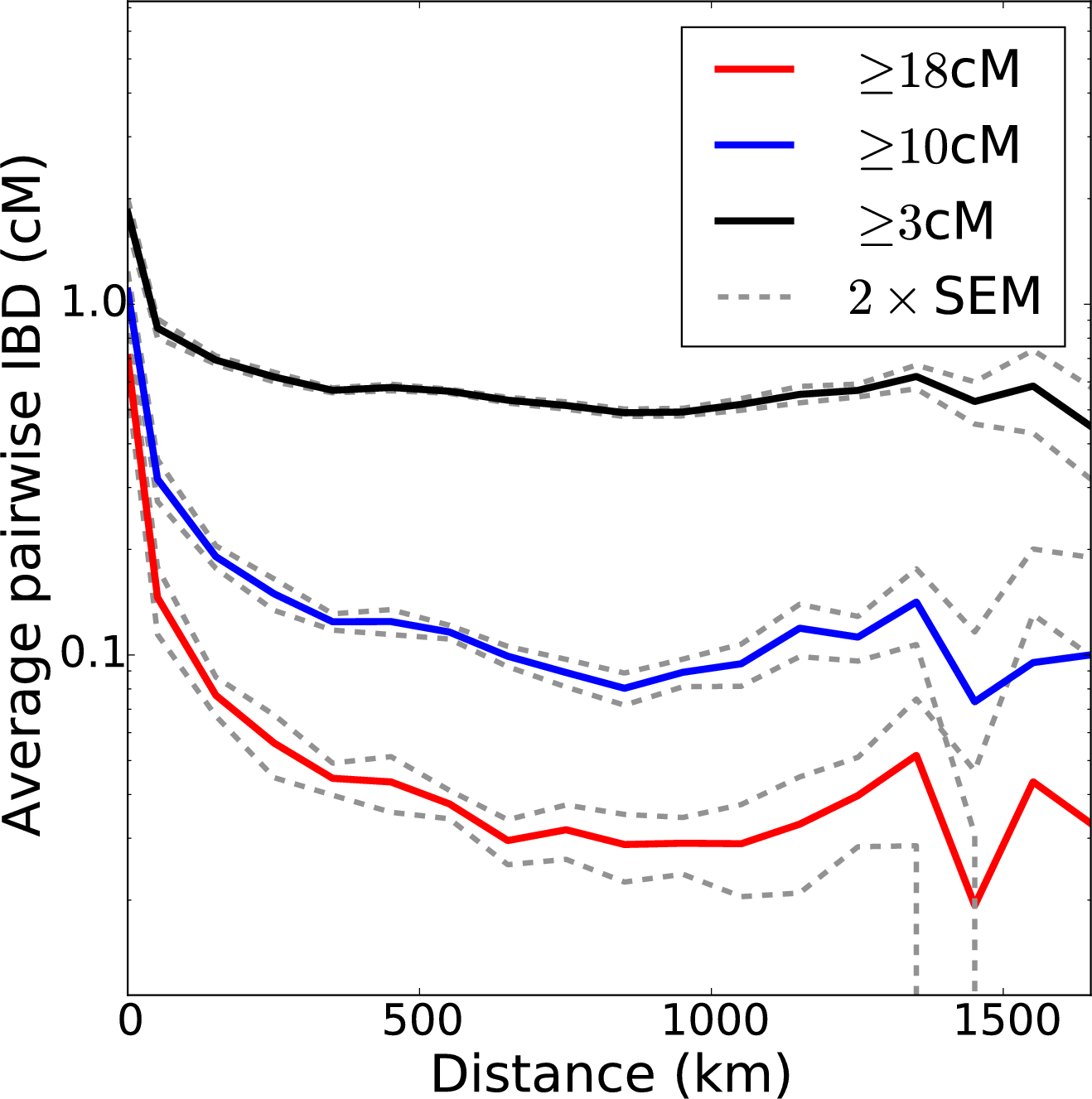

Supplement: S19 Fig — The plot is in log-linear scale, and the dashed lines represent two standard error deviations from the mean for the corresponding curve. (TIF) [file pgen.1006059.s020.tif]

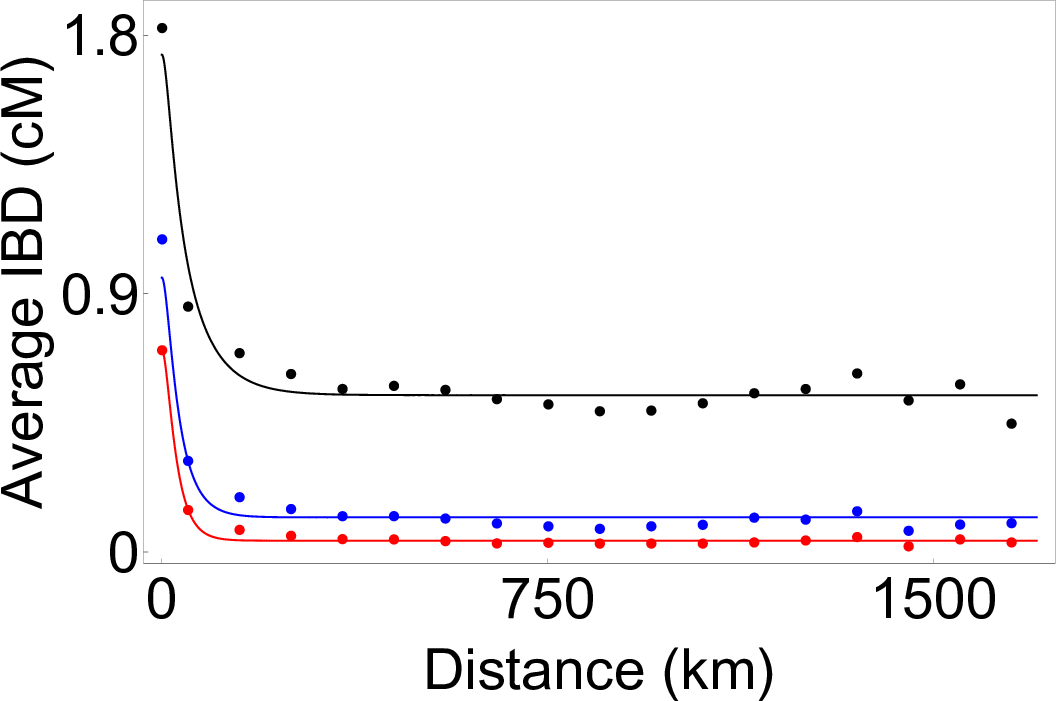

Supplement: S20 Fig — Points represent the data and lines represent the model. (TIF) [file pgen.1006059.s021.tif]

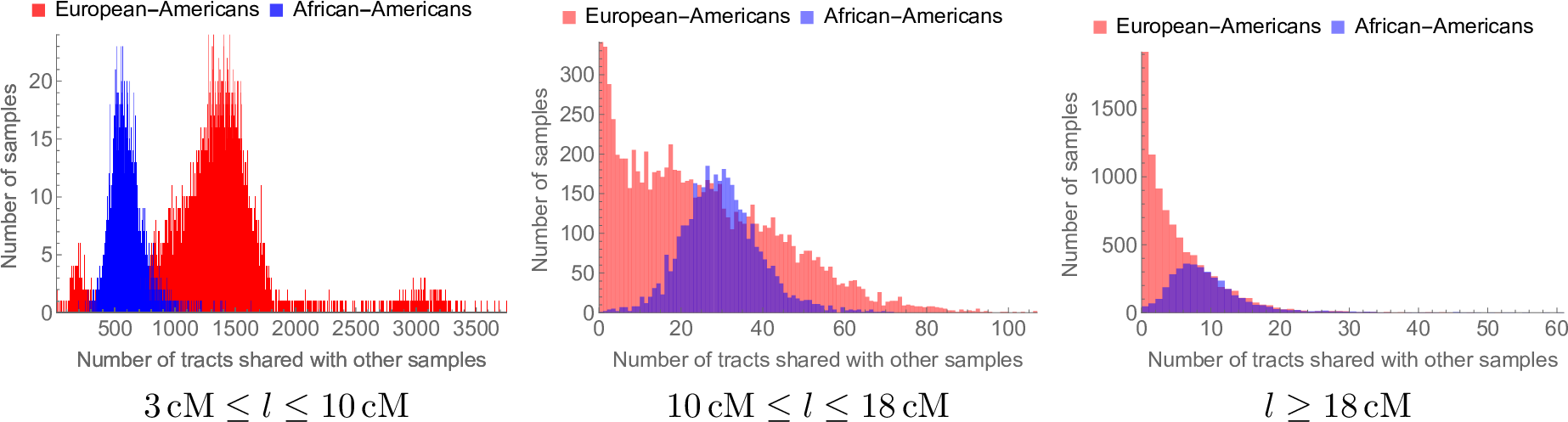

Supplement: S21 Fig — (TIF) [file pgen.1006059.s022.tif]

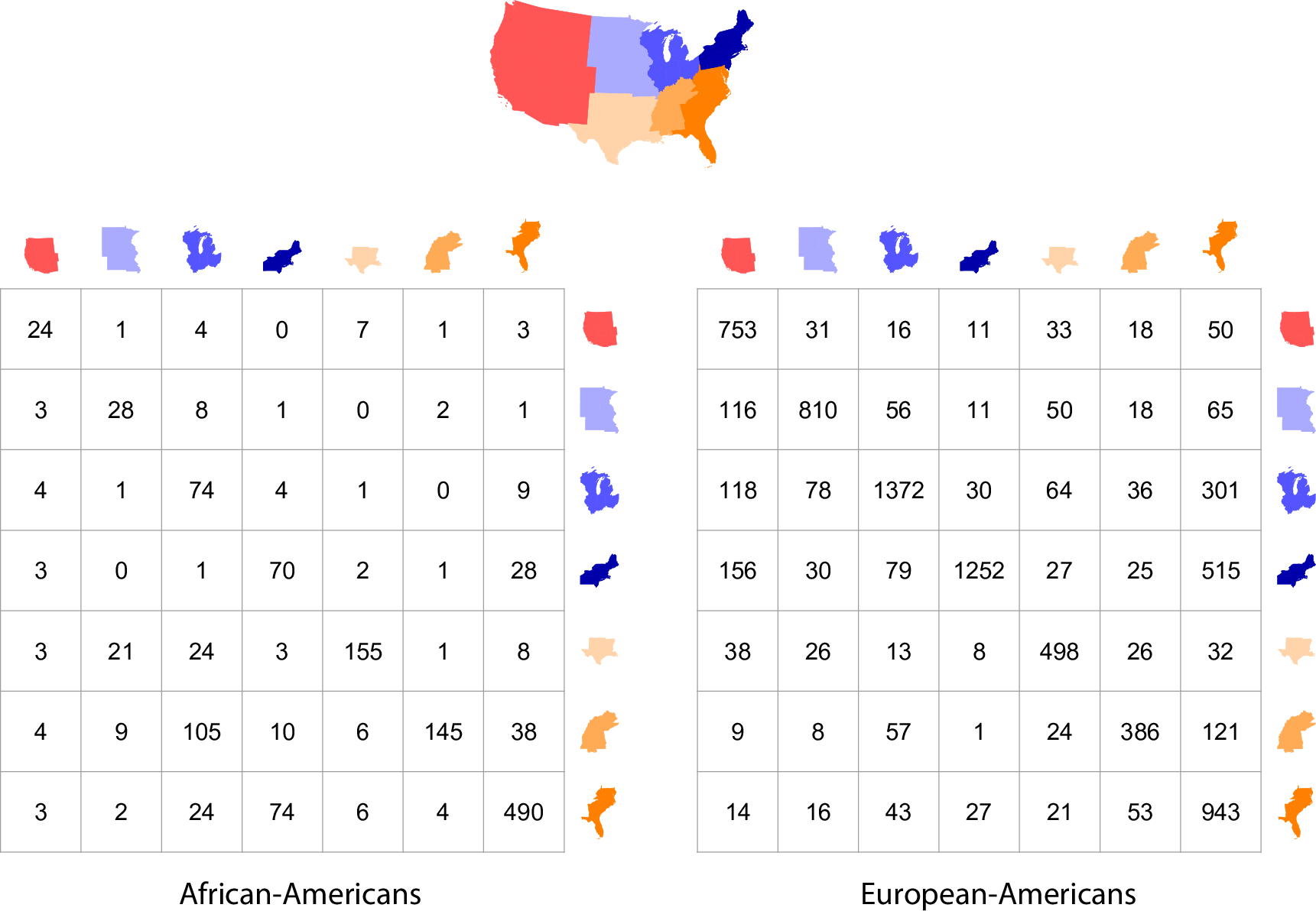

Supplement: S22 Fig — Rows represent regions of birth, and columns represent regions of residence in 2010. (TIF) [file pgen.1006059.s023.tif]
