# Supplementary material for: The Great Migration and African-American Genomic Diversity
Source: PLoS Genet. 2016 May 27;12(5):e1006059. doi: 10.1371/journal.pgen.1006059 (PMC4883799; doi:10.1371/journal.pgen.1006059)
Supplement: S1 Table — (PDF) [file pgen.1006059.s024.pdf]

Cohort	African-American individuals	males / females	Hispanics	locale
HRS	1501	531 / 970	10	contiguous US
SCCS	2128	1131 / 997	N/A	southern US
