# Supplementary material for: The Great Migration and African-American Genomic Diversity
Source: PLoS Genet. 2016 May 27;12(5):e1006059. doi: 10.1371/journal.pgen.1006059 (PMC4883799; doi:10.1371/journal.pgen.1006059)
Supplement: S2 Table — (PDF) [file pgen.1006059.s025.pdf]

census region or division		race	
		African-American	European-American
Northeast		171	1366
East North Central		240	1636
West North Central		62	999
South Atlantic		1249	2027
East South Central		1464	562
West South Central		420	717
West		110	1574
		3716	8881
