# Supplementary material for: The Great Migration and African-American Genomic Diversity
Source: PLoS Genet. 2016 May 27;12(5):e1006059. doi: 10.1371/journal.pgen.1006059 (PMC4883799; doi:10.1371/journal.pgen.1006059)
Supplement: S3 Table — Confidence intervals for selected models inferred using Tracts. Here, ti refers to the time of the ith migration event (in generations ago), and f2EUR refers to the fraction of European admixture in the second migration event. (PDF) [file pgen.1006059.s026.pdf]

birth year	number of individuals
< 1920	57
$1920 - 24$	79
$1925 - 27$	59
$1928 - 30$	78
$1931 - 33$	133
$1934 - 36$	174
$1937 - 39$	177
$1940 - 42$	178
$1943 - 45$	110
$1946 - 48$	147
$1949 - 52$	140
$1953 - 55$	134
> 1955	70
