# Supplementary material for: The Great Migration and African-American Genomic Diversity
Source: PLoS Genet. 2016 May 27;12(5):e1006059. doi: 10.1371/journal.pgen.1006059 (PMC4883799; doi:10.1371/journal.pgen.1006059)
Supplement: S4 Table — Here, ti refers to the time of the ith migration event (in generations ago), and f2EUR refers to the fraction of European admixture in the second migration event. (PDF) [file pgen.1006059.s027.pdf]

Model	Cohort	Parameter	Value	Confidence interval
pp	SCCS	t_1	6.3037	$[6.2316, 6.3918]$
	HRS	t_1	5.8101	$[5.7224, 5.9004]$
pp_xp	SCCS	t_1	9.4954	$[8.5638, 9.8440]$
		t_2	4.4018	$[3.8538, 4.5455]$
		f_2^{EUR}	0.0945	$[0.0736, 0.1015]$
	HRS	t_1	8.3268	$[8.1376, 9.3284]$
		t_2	3.8163	$[3.6980, 4.1718]$
		f_2^{EUR}	0.0986	$[0.0880, 0.1255]$
pxp_xpx	SCCS	t_1	18.0755	$[17.6282, 18.5729]$
		t_2	5.5439	$[5.4747, 5.6168]$
	HRS	t_1	17.4673	$[16.6013, 18.0893]$
		t_2	5.2085	$[5.1249, 5.2913]$
pxp_xpx_xpx	SCCS	t_1	17.8069	$[17.3306, 18.2082]$
		t_2	6.1080	$[6.0304, 6.2183]$
		f_2^{EUR}	0.1233	$[0.1179, 0.1275]$
		t_3	2.2665	$[2.0000, 2.5579]$
	HRS	t_1	17.2778	$[16.4673, 17.8263]$
		t_2	5.7287	$[5.6489, 5.9159]$
		f_2^{EUR}	0.1460	$[0.1363, 0.1505]$
		t_3	2.0000	$[2.0000, 2.6055]$
