# Supplementary material for: The Great Migration and African-American Genomic Diversity
Source: PLoS Genet. 2016 May 27;12(5):e1006059. doi: 10.1371/journal.pgen.1006059 (PMC4883799; doi:10.1371/journal.pgen.1006059)
Supplement: S5 Table — For relateds within HRS, we used the list provided by the Health and Retirement Study. (PDF) [file pgen.1006059.s028.pdf]

161321930	GWAS_0889	NA19625	NA20414	NA19982	NA19983
GWAS_0028	GWAS_2129	NA19700	NA19702	NA19983	NA19985
GWAS_0223	GWAS_2277	NA19701	NA19702	NA20126	NA20128
GWAS_0229	GWAS_2386	NA19703	NA19705	NA20127	NA20128
GWAS_0240	GWAS_1676	NA19704	NA19705	NA20276	NA20277
GWAS_0252	GWAS_2389	NA19707	NA19708	NA20278	NA20279
GWAS_0387	GWAS_2382	NA19713	NA19714	NA20279	NA20282
GWAS_0567	GWAS_2250	NA19713	NA19983	NA20279	NA20284
GWAS_0773	GWAS_1975	NA19713	NA19985	NA20282	NA20284
GWAS_0784	GWAS_1203	NA19714	NA19985	NA20282	NA20302
GWAS_0851	GWAS_1680	NA19818	NA19828	NA20282	NA20313
GWAS_0894	GWAS_1595	NA19819	NA19828	NA20287	NA20288
GWAS_0959	GWAS_1026	NA19834	NA19836	NA20289	NA20290
GWAS_1104	GWAS_1902	NA19835	NA19836	NA20289	NA20341
GWAS_1137	GWAS_1765	NA19900	NA19902	NA20290	NA20341
GWAS_1168	GWAS_1803	NA19901	NA19902	NA20291	NA20292
GWAS_1323	GWAS_1928	NA19908	NA19919	NA20294	NA20295
GWAS_1375	GWAS_2257	NA19909	NA19919	NA20296	NA20297
GWAS_1380	GWAS_1833	NA19914	NA19915	NA20299	NA20300
GWAS_1571	GWAS_1598	NA19916	NA19918	NA20302	NA20313
GWAS_1596	GWAS_2171	NA19917	NA19918	NA20314	NA20316
GWAS_1962	GWAS_2008	NA19920	NA20129	NA20317	NA20319
GWAS_2015	GWAS_2226	NA19921	NA20129	NA20332	NA20333
NA20332	NA20343	NA20342	NA20343	NA20357	NA20358
NA20334	NA20335	NA20344	NA20345	NA20359	NA20360
NA20334	NA20336	NA20344	NA20350	NA20359	NA20363
NA20334	NA20337	NA20346	NA20347	NA20363	NA20364
NA20335	NA20336	NA20347	NA20363		
NA20336	NA20337	NA20356	NA20358		
